# Accelerated full-thickness skin wound tissue regeneration by self-crosslinked chitosan hydrogel films reinforced by oxidized CNC-AgNPs stabilized Pickering emulsion for quercetin delivery

**DOI:** 10.1186/s12951-024-02596-0

**Published:** 2024-06-08

**Authors:** Garima Sharma, Jomon George Joy, Ashish Ranjan Sharma, Jin-Chul Kim

**Affiliations:** 1 Department of Biomedical Science & Institute of Bioscience and Biotechnology, Kangwon National University, Chuncheon, 24341 Republic of Korea; 2 Institute for Skeletal Aging & Orthopedic Surgery, Hallym University-Chuncheon Sacred Heart Hospital, Chuncheon-si, 24252 Gangwon-do Republic of Korea

**Keywords:** Chitosan hydrogel film, Pickering emulsion, Dialdehyde cellulose nanocrystal, Silver nanoparticles, Quercetin, Wound healing

## Abstract

**Background:**

The non-toxic self-crosslinked hydrogel films designed from biocompatible materials allow for controlled drug release and have gathered remarkable attention from healthcare professionals as wound dressing materials. Thus, in the current study the chitosan (CS) film is infused with oil-in-water Pickering emulsion (PE) loaded with bioactive compound quercetin (Qu) and stabilized by dialdehyde cellulose nanocrystal-silver nanoparticles (DCNC-AgNPs). The DCNC-AgNPs play a dual role in stabilizing PE and are involved in the self-crosslinking with CS films. Also, this film could combine the advantage of the controlled release and synergistic wound-healing effect of Qu and AgNPs.

**Results:**

The DCNC-AgNPs were synthesized using sodium periodate oxidation of CNC. The DCNC-AgNPs were used to stabilize oil-in-water PE loaded with Qu in its oil phase by high speed homogenization. Stable PEs were prepared by 20% v/v oil: water ratio with maximum encapsulation of Qu in the oil phase. The Qu-loaded PE was then added to CS solution (50% v/v) to prepare self-crosslinked films (CS-PE-Qu). After grafting CS films with PE, the surface and cross-sectional SEM images show an inter-penetrated network within the matrix between DCNC and CS due to the formation of a Schiff base bond between the reactive aldehyde groups of DCNC-AgNPs and amino groups of CS. Further, the addition of glycerol influenced the extensibility, swelling ratio, and drug release of the films. The fabricated CS-PE-Qu films were analyzed for their wound healing and tissue regeneration potential using cell scratch assay and full-thickness excisional skin wound model in mice. The as-fabricated CS-PE-Qu films showed great biocompatibility, increased HaCat cell migration, and promoted collagen synthesis in HDFa cells. In addition, the CS-PE-Qu films exhibited non-hemolysis and improved wound closure rate in mice compared to CS, CS-Qu, and CS-blank PE. The H&E staining of the wounded skin tissue indicated the wounded tissue regeneration in CS-PE-Qu films treated mice.

**Conclusion:**

Results obtained here confirm the wound healing benefits of CS-PE-Qu films and project them as promising biocompatible material and well suited for full-thickness wound healing in clinical applications.

**Graphical Abstract:**

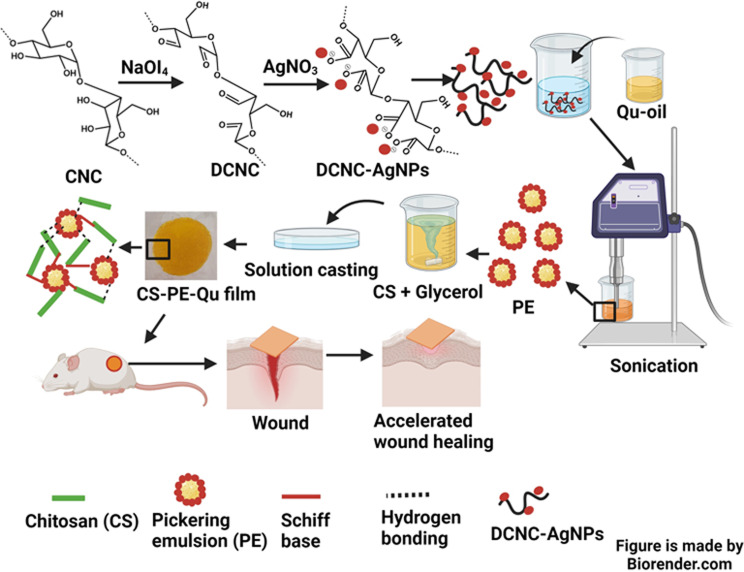

**Supplementary Information:**

The online version contains supplementary material available at 10.1186/s12951-024-02596-0.

## Introduction

Since wound healing is a complex phenomenon, late wound healing and hypertrophic scarring are key challenges that impact the quality of life [1, 2]. Thus, the development of novel biocomposite materials with great properties to repair wounds and anti-scar is requisite. The wound-healing effects of quercetin (Qu) have been reported previously [3] and can be used as a topical therapy to increase fibroblast proliferation, reduce immune cell infiltration, and modulate fibrosis signaling pathways [4]. However, its low solubility in water and biological fluids reduces its bioavailability and delivery to wounds. Moreover, Qu can only penetrate the dermal layer. Hence, to achieve consistent and effective transdermal delivery of Qu at therapeutic concentrations, it is imperative to employ an appropriate carrier, such as Pickering emulsions (PEs). This choice is crucial as it should enable a substantial payload capacity and facilitate optimal skin penetration.

The PEs, stabilized by solid particles, are less hazardous and more stable than surfactant-stabilized ones and are known for good skin penetration and a high payload capacity [5]. It has been reported that water-in-oil (W/O) PEs stabilized by adsorbed silica particles increased skin permeation of hydrophilic caffeine compared to a classical surfactant-based emulsion [6]. Interestingly, oil-in-water (O/W) PEs also increased skin permeation of hydrophobic trans-retinol permeate [7]. Thus, PEs are suitable for topical medication delivery of both hydrophobic and hydrophilic compounds due to their tunable characteristics, surfactant-free nature, enhanced stability, and capacity to vary skin delivery rates and absorption sites [8]. Since O/W PEs enhance hydrophobic drug penetration, adding Qu to them can overcome the topical administration constraints with Qu. Nanoparticles (NPs) from silica, gold, palladium, silver (Ag), iron oxide, titanium dioxide, and zinc oxide have been used to synthesize thermodynamic and kinetically stable PEs [9–16]. Silver nanoparticles (AgNPs) are renowned for their low toxicity, excellent anti-fouling, slow-release, and wound healing characteristics. Thus, the production of AgNP-stabilized PEs containing Qu may provide a synergistic wound healing effect.

Nevertheless, topical application of PEs requires a dressing matrix, such as films. Films employing PEs serve two reasons, first, the hydrophilic matrixes lack water vapor barrier properties which can be improved by evenly distributed oil droplets throughout the matrix [17]. Failure to disperse might cause discontinuities, which may increase water vapor diffusion, counteracting the desired effect. Second, an oil phase on polymeric films can transport hydrophobic active chemicals, generating active films and coatings, such as those with essential oils [18]. The release pattern of active components in films affects their effectiveness, depending on factors like matrix crystallinity and surfactant/matrix interactions in materials based on conventional emulsions [19]. However, thicker interface layers of PEs act as encapsulation systems, slowing release and extending activity time, making them better for the controlled release of active compounds [18].

Biodegradability, biocompatibility, and hemostatic properties make chitosan (CS) wound dressing films a better choice to serve as a PEs matrix [20–22]. However, due to its poor mechanical properties, CS is reinforced with other biopolymers to improve its mechanical and physical properties while retaining its biocompatibility. Biodegradable cellulose nanocrystal (CNC) has unique optical, rheological, and mechanical properties [23]. The CNCs have a high length-to-width aspect ratio (10 to 100), strongly linked network structure, remarkable transparency, and exceptional mechanical strength and stiffness making them ideal reinforcement agents [24]. Previous research has demonstrated that CNCs and CS can create bio-nanocomposite films with improved mechanical strength and water vapor resistance [25]. CNC particles tend to form agglomerates when coupled with CS in solutions, restricting their use in CS films [26, 27]. Gao et al. showed that dialdehyde CNC (DCNC) may be employed as a reinforcing agent to make CS films using a simple solution-casting approach [28]. Studies showed that DCNC can reduce and stabilize AgNPs in a biocompatible way [29], which could be used to fabricate biocompatible films.

**Scheme 1:**
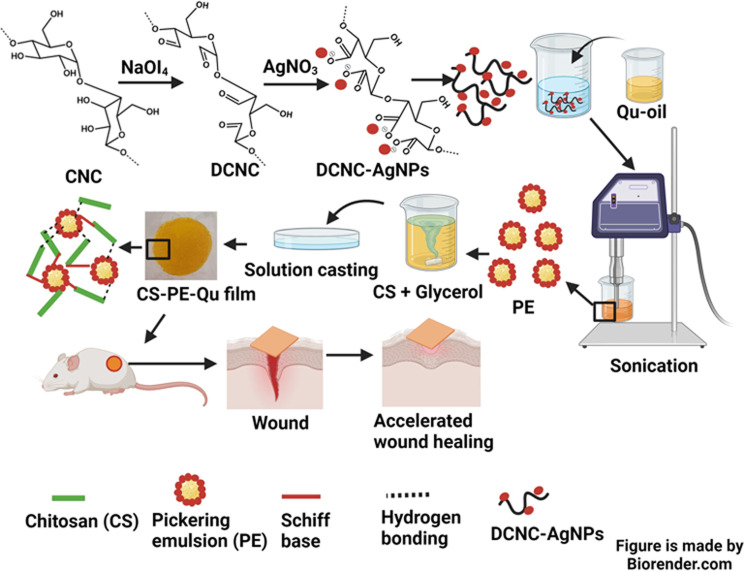
The workflow for the fabrication of chitosan films reinforced with DCNC-AgNPs-stabilized quercetin-loaded Pickering emulsion and its wound healing properties. Created with BioRender.com

We hypothesize that the Pickering emulsion will serve as an efficient carrier for Qu, leading to improved bioavailability and controlled release in wounds. Further, we anticipate that the Pickering emulsion, stabilized by AgNPs will provide additional wound healing effects. Additionally, we predict that the CS could be a suitable matrix for the application of AgNPs-stabilized Qu-loaded PEs. This would provide the dual benefit of not only stabilizing an O/W emulsion encapsulating hydrophobic Qu in an emulsifier-free formulation but also allowing the fabrication of AgNPs functionalized biocompatible CS films. Thus, in this study, we developed CS films reinforced with Qu-loaded PE that are stabilized with DCNC-AgNPs (Scheme 1), which has not been studied yet.

## Methods

### Materials

Chitosan (CS; low molecular weight (< 100 kD); deacetylation ≥ 75%), NaOH, quercetin (Qu; ≥95% (HPLC)), PBS buffer, acetic acid, calcofluor white and ethylene glycol were purchased from Sigma-Aldrich. Cellulose nanocrystal (CNC) was purchased from CelluForce NCC™ (Canada). Olive oil was bought commercially. Sodium periodate and isoflurane were purchased from JUNSEI, Korea, and Hana Pharm. co. ltd, Korea, respectively. DMEM media and RPMI media were purchased from Gibco, UK.

### Synthesis and characterization of DCNC

Periodate oxidation of CNC was performed as reported elsewhere with minor modifications [28, 30]. Typically, 100 mg of cellulose was overnight soaked in DDW water under stirring. 100 mg of NaIO_4_ was then added to the reaction solution at 60 °C. The oxidation time was optimized by performing different sets of experiments at varying times. At the end of the reaction, ethylene glycol was added to the reaction vessel to quench the residual periodate. The reaction solution was dialyzed (MWCO 3.5 kD) for 3 days to remove unreacted chemicals. The solution was then lyophilized for further experiments. The aldehyde content was determined by the hydroxylamine hydrochloride method [31]. The DCNC was further characterized using proton nuclear magnetic resonance (^1^H NMR), Fourier transform infrared spectroscopy (FT-IR), and X-ray diffraction (XRD) (Supplementary information).

### Synthesis of DCNC-AgNPs

DCNC-AgNPs were prepared as reported elsewhere with slight modifications [30]. DCNC and AgNO_3_ were used for the spontaneous *in-situ* production of DCNC-AgNPs nanocomposite in a chemical-free reducing environment at room temperature (25 °C). In brief, 100 mL of 0.1% DCNC suspension was mixed with 1 mM of AgNO_3_ and stirred for 5 min, and placed on a rotary shaker in dark overnight to enhance the absorption of Ag^+^. Then, the pH of the solution was then adjusted to 11 using 0.1 M NaOH in an ice-water bath and was transferred immediately to room temperature to initiate the reaction. The synthesized DCNC-AgNPs were centrifuged at 11,000 rpm for 30 min and washed with DDW three times to remove unreacted Ag ions or unattached AgNPs. The finally obtained DCNC-AgNPs were redispersed in Milli-Q water for further experiments. The as-synthesized DCNC-AgNPs were characterized using UV-Vis absorbance, dynamic light scattering (DLS), transmission electron microscopy (TEM), FT-IR, XRD, and X-ray photoelectron spectroscopy (XPS) (Supplementary information).

### Fabrication of PEs

The Qu-loaded PE stabilized by DCNC-AgNPs was fabricated using the method described elsewhere [13]. The oily phase was created by dissolving Qu into olive oil and determining the maximum Qu concentration soluble in the oil phase. For this, an excess amount of Qu was added to olive oil and stirred at 50 °C for 1 h followed by sonication for 10 min with 10s on/10s off cycle at 40% frequency in an ice bath, followed by centrifugation at 10,000 rpm for 30 min to precipitate out undissolved Qu and ensure maximum Qu solubility in olive oil. Then, the oily phase was injected dropwise by syringe to the DCNC-AgNPs aqueous phase during homogenization with a high-speed homogenizer at a speed of 12,000 rpm and room temperature for 10 min. The emulsions were then placed in airtight glass vials and kept at room temperature until further research. The PEs were visualized using an optical microscope (Olympus CX31, Techsan Microsystem) and a confocal laser scanning microscope (Supplementary information).

### Fabrication of chitosan films reinforced with PE (CS-PE films)

The PE-loaded CS films were prepared by adding 0% (v/v), 25% (v/v), 50% (v/v), and 75% (v/v) of PEs, separately, to low molecular weight CS solution in 2% acetic acid. The final CS weight in all the solutions was constant, i.e., 2% w/v. The solutions of PE and CS were lyophilized and the crosslinking between the PE and the amino group of CS was identified using FT-IR. Further, to impart flexibility in CS films, 2.5%, 10%, 25%, and 35% glycerol was added as a plasticizer to the PE and CS mixed solution and stirred for 15 min before drop casting on polyethylene Petri dishes. The dishes were dried in an oven at 40 °C for 24 h. The amount of CS and the mass of suspension applied to the Petri dishes were kept constant in all the films.

### Mechanical parameters

Films were cut into 20 mm x 15 mm strips and were mechanically tested for mechanical parameters using a Universal test machine (Instron, 3367, U.S.A.). The load cell was 100 N, the test grip was 25 N and the ramp rate was 10 mm/min. The modulus and elongation at break were determined.

### Porosity

The porosity of the films was assessed using the water displacement method as described in a previous study [32]. The films were cut into little pieces of 1 × 1 cm, and their respective weights were recorded. The film was transferred into a Petri dish that contained 5 mL of water and subsequently sealed. The film was immersed in an aqueous solution for 24 h, until it achieved a state of equilibrium. Subsequently, the film was extracted and subsequently subjected to a second weighing procedure. The equation presented herein was utilized for the determination of the porosity of the films.

$$\text{P}\text{o}\text{r}\text{o}\text{s}\text{i}\text{t}\text{y} \left({\%}\right)= \frac{(W2-W1)}{\left(W2\right)}{\times} 100$$

Here W1 is the initial weight of the film before immersing in the water, and W2 represents the weight of the film after removing from water.

### Swelling ratio

The swelling ratio or water absorption capabilities of the films were assessed by incubating the films (1 × 1 cm) at room temperature in a 0.9% saline solution. At specific time intervals, the films that had been soaked were removed from the medium and their weights were measured to determine the extent of swelling. This was done by removing any extra water from the film’s surface using filter paper. After being weighed, the films were returned to the saline medium. The proportion of swelling was determined using the following calculation [33]:

$$\text{S}\text{w}\text{e}\text{l}\text{l}\text{i}\text{n}\text{g} \left({\%}\right)= \frac{(Ws-Wi)}{Wi}{\times} 100$$

Where W_s_ represents the weight of the soaked film and W_i_ indicates the weight of the dry film initial.

### pH dressing

The dressing pH of the films was determined using the digital pH meter for 24 h and 48 h respectively. Each of the films was soaked in 0.9% saline solution until it reached the equilibrium. Afterward, the film was removed and the same solution was used for the determination of the dressing pH. The experiment was repeated three times and average values were recorded.

### Water vapour transmission rate

The Water vapor transmission rate (WVTR) was determined as described previously [33]. In summary, the film samples were affixed to the aperture of custom-designed containers, which were filled with 15 mL of distilled water, reaching a height of 1.5 cm from the underside of the film. The cups were precisely weighed and subjected to incubation at a temperature of 25 °C. The cups were subjected to a second weighing 24 h later, and the resulting data was utilized to construct a graph illustrating the relationship between the rate of mass change and the elapsed time for each film.

$$\text{W}\text{V}\text{T}\text{R} \left(\text{g}{m}^{-2}{day}^{-1}\right)= \frac{M {\times} 24}{T {\times} A}$$

Where M indicates the change in weight, T for the time duration of the weight change, and A is the area of the film used (m^2^).

### Quercetin and silver ion release

The film was submerged in 50 mL of pH 7.4 PBS buffer supplemented with 1% Tween 80 to maintain the sink condition. After 0, 1, 2, 3, 4, 6, 8, 10, 24, and 48 h, 1 mL of the solution was removed and replaced with the same volume of PBS buffer. The sample solutions were mixed with 20% methanol, kept on a rotary shaker for 2 h, and centrifuged at 5000 rpm for 20 min. The supernatant was collected and measured spectrophotometrically at 370 nm. The Qu was calculated using a standard curve of Qu in methanol. For the Ag^+^ release assay, the films were submerged in 50 mL of pH 7.4 PBS buffer. After 24 and 48 h, 1 mL of the solution was removed and replaced with the same volume of PBS buffer. The samples were diluted with 10% HNO_3_ and analyzed for Ag^+^ discharge using Inductively coupled plasma mass spectrometry (ICP-MS) [34, 35].

### In vitro skin permeation study

The skin permeation study was performed in accordance with previous literature with slight modifications [36]. The Strat-M® membranes (25 mm in diameter and 300 μm thickness, Merck Millipore (Molsheim, France)) attaching the CS-Qu, CS-blank PE, and CS-PE-Qu films were put on the receptor chamber of a Franz diffusion cell and the donor cell was fixed on the membranes-mounted receptor chamber using a clamp. The physiological solution, i.e., 2.38 g of Na_2_HPO_4_, 0.19 g of KH_2_PO_4_, and 9 g of NaCl into 1 L of Milli-Q water (final pH = 7.35) containing 1% Tween 80 [37, 38], was added in the receptor chamber as the receiving liquid to reproduce the saline concentration of the bloodstream so that the medium came into contact with the membranes. The assemblies were gently stirred for 48 h and samples were withdrawn at pre-determined time points supplementing an equal amount of fresh isothermal liquid after withdrawal. The collected samples were analyzed for Qu and Ag^+^ contents using HPLC and ICP-MS, respectively.

### Cell cytotoxicity assay

The immortalized adult human skin keratinocyte (HaCaT) and Human Dermal Fibroblasts (HDFa) cell lines were obtained from the American Type Culture Collection (ATCC; Manassas, VA, USA). Both the cell lines were maintained in Dulbecco’s Modified Eagle’s Medium (DMEM) supplemented with 10% fetal bovine serum and 1% streptomycin/penicillin solution. Cells were cultured at 37 °C, 5% CO_2_, and 95% humidity incubator.

The Cell Counting Kit-8 (CCK-8) was used to observe the viability of HaCat and HDFa cells after treatment with CS film, CS-Qu film (prepared by physically mixing an equal amount of loaded Qu), CS-blank PE film (prepared by adding blank PE stabilized by AgNPs-DCNC), and CS-PE-Qu film (prepared by adding Qu-loaded PE stabilized by AgNPs-DCNC). The untreated cells were used as a negative control. In brief, to prepare treatment media, each film (1 cm x 1 cm) was incubated separately in 2.5 mL (extract 1), 5 mL (extract 2), and 10 mL (extract 3) of serum-free DMEM media for 24 h. The media was collected and sterilized using a syringe filter. HaCat and HDFa cells (5 × 10^3^) were seeded into each well of a 96-well plate for 24 h, followed by replacing the media with treatment DMEM media containing 1% FBS and incubating for 24 h. After the incubation time, the treatment media were replaced with DMEM media containing 10% of CCK-8 reagent and further incubated for 4 h at 37 °C. The optical density was then measured at 450 nm using a microplate reader. The % cell viability was calculated as the equation mentioned below. The experiment was performed in triplicate [39].

$$Cell\,viability \left(\%\right)=\left(\frac{{A}_{450}\,of\,treated\,cells}{{A}_{450}\,of\,control\, cells}\right){\times}100$$

Where, A_450_ is absorbance taken at 450 nm.

### Cell scratch assay

The confluent HaCaT and HDFa cells were seeded in 24 well plates at the 1 × 10^5^ cell/well density. After incubation for 24 h, scratches were induced using sterile 200 µL pipette tips in all the monolayers of cells across the diameter of the wells. The media was aspirated, and the cells were washed twice. The treatment DMEM media containing extract of CS films, CS-Qu, CS-blank PE, and CS-PE-Qu films (as prepared above) were added to the wells. The plates were incubated at 37 °C. The images of each well were taken at 0 h, 24 h, and 36 h of treatment using a bright field microscope [30].

### Protein extract and western blotting

The HDFa cells were treated with CS, CS-Qu, CS-blank PE, and CS-PE films for 48 h, as described above. After treatment, the medium was removed followed by washing with ice-cold PBS, and treated for 15 min with lysis cocktail buffer containing NaO, NaF, phosphatase, and protease inhibitor (Roche Diagnostics, Germany). All cell lysates were obtained after 15 min of centrifugation at 14,000 rpm. A protein assay kit (BioRad, USA) was used to measure protein in samples per the manufacturer’s procedure. Gel electrophoresis was performed on 10% SDS-polyacrylamide gel with equal protein loading for each sample. Separated proteins were transferred to PVDF membranes (Millipore, USA). Incubated blots using 1:1000 dilutions of a primary antibody of Col1α (Santa Cruz; Sc 25,974) in 1% BSA. After washing three times with TBST (10 mM Tris HCl, 50 mM NaCl, 0.25% Tween 20), blots were incubated with a horseradish peroxidase-conjugated goat secondary antibody at 1:5000 dilution (Jackson Immunoresearch, USA) and rinsed twice with TBST. Finally, chemiluminescence (ECL) reagents (BioNote Inc., Korea) photographed the bands. Antibody against *β*-actin used as a loading control. Western blot densitometry was also done (Fusion FX, Vilver Lourmat, France) [40].

### Animals

To evaluate the wound healing efficiency of the films, the BALB/c female mice (*n* = 30) weighing between 20 g and 30 g were used. The animals were divided into five groups (*n* = 6) and were kept in suitable polypropylene boxes housed at 22 ± 2 °C and 60 ± 15% relative humidity with a 12 h light/dark cycle. The rodents were fed with standard laboratory chow and water *ad libitum*. All animal experiments comply with the ARRIVE guidelines and were carried out in accordance with the National Research Council’s Guide for the Care and Use of Laboratory Animals and according to the Institutional Animal Care and Use Committee (IACUC) of Kangwon National University (KIACUC-KW-220711-3) as directed by Ministry of Agriculture, Food and Rural Affairs [Animal protection act] and Ministry of Food and Drug Safety [Laboratory Animal Act], Republic of Korea.

### Hemocompatibility study

An in vitro hemolysis assay was used to determine the hemocompatibility of CS films, CS-Qu, CS-blank PE, and CS-PE-Qu films. In the fresh anticoagulant whole blood from a healthy rodent, prepared by adding 3.8% of Na-citrate anticoagulant solution, was centrifuged and repeatedly washed with PBS to obtain the red cells. The films were separately added to the diluted red cells (5% (v/v) with PBS solution). and incubated for 4 h at 37 °C. The red cell suspensions in PBS without film and in 0.1% Triton X-100 were taken as negative control and positive controls, respectively. Post-incubation, the red cell suspension tubes were then subjected to centrifugation at 10,000 rpm for 10 min, and the absorbance of the supernatant was recorded at 545 nm. The hemolysis rate was calculated as follows [41]:

$$Hemolysis\,rate \left(\%\right)=\frac{\left({OD}_{s}- {OD}_{n}\right)}{\left({OD}_{p}- {OD}_{n}\right)}{\times} 100$$

Where, ODs, ODn, and ODp represented the absorbance of supernatants of the samples, the negative control, and the positive control, respectively.

### Wound healing assay

The wound healing assay was performed as described elsewhere with modifications [42, 43]. The mice were anesthetized with isoflurane. To establish the surgical area, the dorsal hair was removed using an electric hair clipper. Full-thickness excisional skin wounds were generated on the dorsal side of BALB/c mice using a biopsy punch with a diameter of 4 mm. Over a period of 10 days, the control group received dressing only with medical gauze. In contrast, the wounds in other groups were covered with respective CS films, CS-Qu, CS-blank PE, and CS-PE-Qu films and covered by transparent film dressings. The films were changed daily, followed by daily sealing of the wound using commercial film dressing. The wounds were photographed on days 0, 3, 7, and 10. The wound healing ratio is calculated as follows:

$$Wound\,closure\,ratio \left(\%\right)=\left(\frac{{S}_{0}-{S}_{n}}{{S}_{0}}\right){\times} 100$$

Where S_0_ is the initial size of the wound and S_n_ is the size of the wound at a given time point.

### Microscopic evaluation

On day 10, the mice were euthanized by CO_2_ asphyxiation and the wound areas were excised for microscopic evaluation. Normal skin sections were also acquired as a positive control. The samples were immersed in a solution of 10% neutral buffered formalin, subjected to standard processing procedures, embedded in paraffin, cut into sections measuring 5 μm in thickness, and subsequently stained using hematoxylin and eosin. The stained slides were imaged using Slide Scanner (KF-PRO-005-ex) with K-viewer software.

### Statistical analysis

Graphpad prism (Version 5.0) was used to analyze the data by two-tailed Student’s t-test statistically. P value < 0.05 was taken as statistically significant.

## Results and discussion

### Synthesis and optimization of DCNC

To enable the oxidation of CNC, periodate was chosen because of its specific targeting of the C2 and C3 positions. This led to the transformation of hydroxyl groups into aldehyde groups at the C2 and C3 positions by opening the glucose ring. The aldehyde concentration in DACNF can be increased by modulating the reaction time and/or the periodate amount. In the present study, we kept the periodate amount constant and increased the reaction time to achieve maximum dialdehyde content. It was found that increasing the reaction time from 2 h to 8 h was also found to increase the aldehyde concentration, which further remained constant at 10 h, possibly due to the saturation of the reaction. DCNC had the highest aldehyde content of 6.5 mM/g after 8 h (Supplementary Table S1). Thus, 8 h of reaction time was further selected for AgNPs synthesis.

#### Characterization of DCNC

**Fig. 1:**
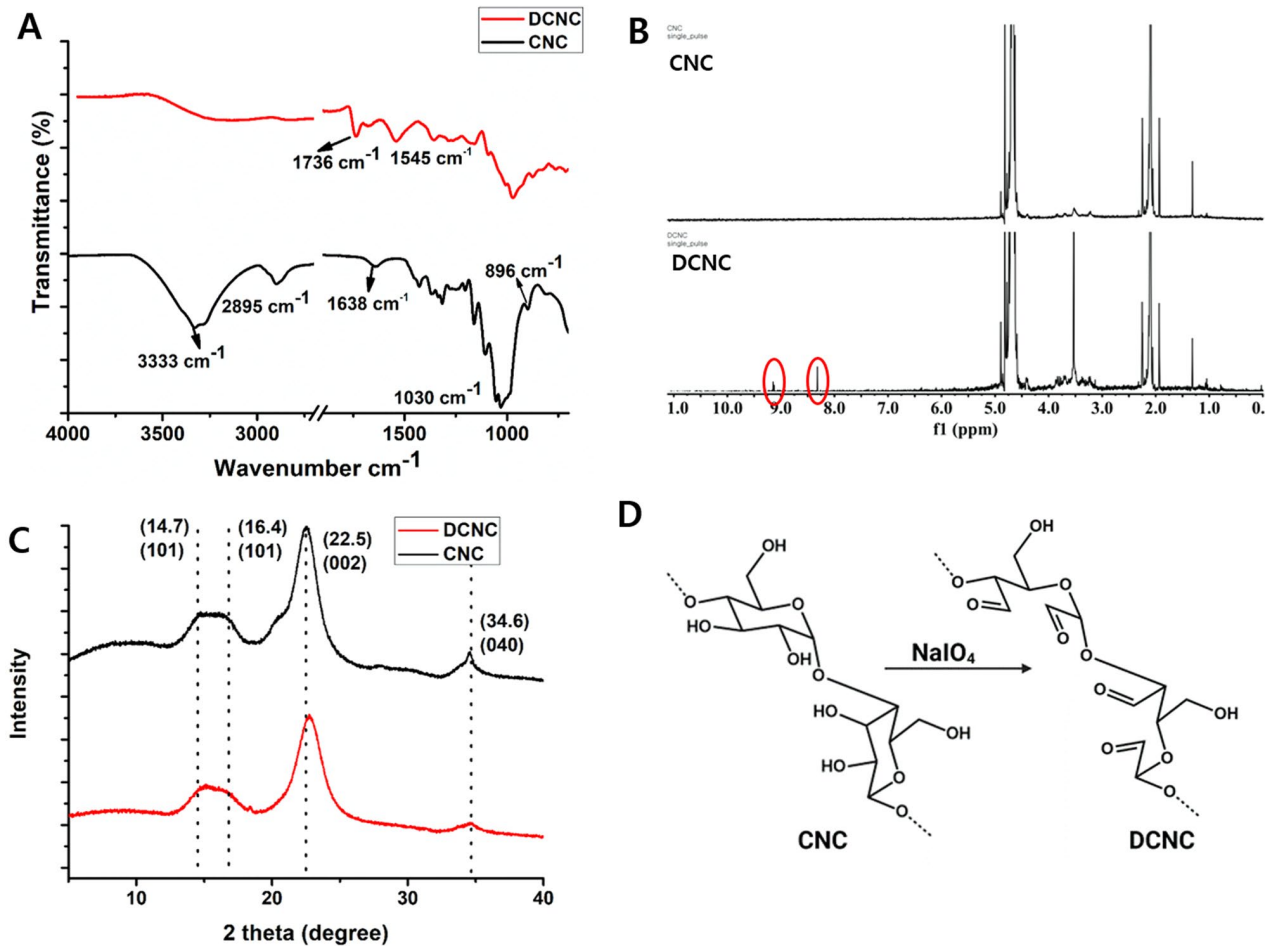
Characterization of DCNC and CNC (**A**) FT-IR spectrum (**B**) ^1^H NMR spectrum (**C**) XRD spectrum and (**D**) Schematic oxidative reaction of CNC

The DCNC was further characterized using TEM, DLS, FT-IR, NMR, and XRD. The size and morphology of DCNC are shown in supplementary Fig. S1. The FT-IR spectrum supports the periodate oxidation of CNC (Fig. 1A). The FT-IR spectra of CNC showed characteristic absorption peaks at 3333 cm^− 1^ for -O-H stretching vibration, at 2895 cm^− 1^ symmetric C-H stretching vibrations, at 1638 cm^− 1^ for –CO symmetric and asymmetric stretching vibration, and 1030 cm^− 1^ for C = O stretching vibration of pyranose ring. The characteristic absorption peak of the *β*-glycosidic bond in CNC attributing to the C-H scissor vibration appeared at 896 cm^− 1^, indicating the pyranose skeleton of CNC with a *β*-glucosidic bond. Most of these peaks remained almost unchanged in the FT-IR spectrum of DCNC. However, the sharpness of the peaks was reduced after oxidation, possibly due to the decrease in the crystallinity of CNC [44, 45]. Moreover, a new small absorption peak appeared at around 1736 cm^− 1^ in the FT-IR spectrum of DCNC, attributing to the characteristic absorption peak of the carbonyl group (C = O) of aldehyde moiety. The stretching vibration band of O-H at around 3333 cm^− 1^ was narrowed and broadened in the FT-IR spectrum of DCNC, possibly due to the involvement of the hydroxyl group in the oxidation process. The peak at 2895 cm^− 1^ in CNC was also reduced in DCNC. Previous studies have indicated that the hydrogen bonds in the cellulose type I structure are arranged axially. The oxidation process using periodate; however, would disturb the existing distribution of hydrogen bonds and generate numerous disordered hydrogen bonds. Consequently, this would result in the widening of the stretching vibration of O-H and C-H [28].

The ^1^H NMR spectra of oxidized DCNC showed the appearance of chemical signals at around 8.31 ppm and 9.1 ppm are assigned to aldehyde hydrogen which confirmed the successful dialdehyde CNC formation (Fig. 1B). The peak for aldehyde hydrogen was small possibly due to the presence of a very small number of free aldehydes. Another peak around 3.5 ppm was also observed that could be assigned to the hydrogen atoms bonded with carbons at the polymer matrix. This spectrum is comparable with that in the literature [46].

Figure 1C illustrates the X-ray diffraction patterns of CNC and DCNC. Both samples exhibit characteristic cellulose type I structures, as evidenced by prominent 2θ diffraction angles around 14.7°, 16.4°, 22.5°, and 34.6° corresponding to the 101, 101, 002, and 040 crystalline planes, respectively. Notably, the crystal structure of DCNC remained unchanged following oxidation, consistent with previous findings [28]. Minor reduction in the crystallinity index of CNC (i.e., 49.67%) was observed after periodate oxidation (i.e., 46.46%), indicating that although periodate oxidation leads to the ring-opening of glucopyranose, the ordered structure of cellulose molecules is not highly disrupted **(**the scheme is shown in Fig. 1D).

#### Synthesis and characterization of DCNC-AgNPs

**Fig. 2:**
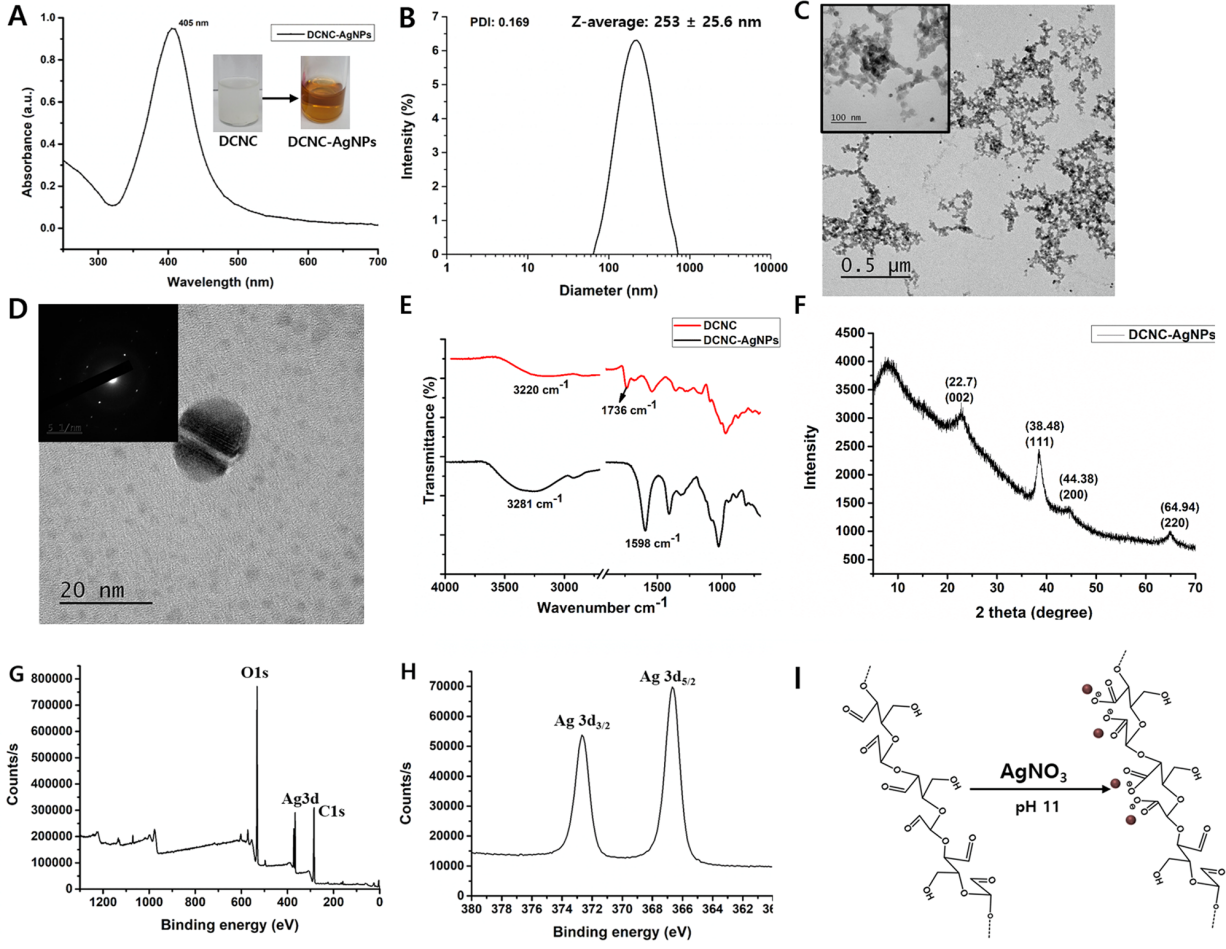
Characterization of DCNC-AgNPs (**A**) UV-Vis spectra (digital photo of DCNC and synthesized DCNC-AgNPs as inset) (**B**) DLS spectrum (**C**) TEM images (**D**) HR-TEM and SAED pattern (inset) (**E**) FT-IR spectrum (**F**) XRD spectrum (**G**)&(**H**) XPS spectrum. (**I**) Schematic reaction of DCNC-AgNPs synthesis

A visibly distinctive dark brown color of AgNPs was observed after the reaction between DCNC and AgNO_3_ (Fig. 2A (inset)). The UV-vis spectrum of DCNC is shown in Supplementary Fig. S1. The UV-Vis absorption spectra of DCNC-AgNPs also showed a peak at 405 nm which is a characteristic peak of AgNPs due to the excitation of surface plasmon resonance (SPR) vibrations of AgNPs synthesized during the reaction (Fig. 2A) [47], suggesting the presence of AgNPs in the reaction solution. Since no additional reducing and stabilizing agent was added to the reaction solution, it can be concluded that the dialdehyde groups present in DCNC assisted in the formation and stabilization of AgNPs [29]. Further, we elucidated the hydrodynamic size of DCNC-AgNPs using DLS, which was 253 ± 25.6 nm and 0.169 polydispersity index (PDI) (Fig. 2B). The hydrodynamic size of DCNC was also around 397.5 nm and the TEM images of DCNC showed a small fibrous network of varying length and diameter (Supplementary Fig.S1. However, the TEM images showed the presence of spherical DCNC-AgNPs having a size range of less than 10 nm on the fibrous network of DCNC (Fig. 2C). Moreover, the deposition of AgNPs on the surface of DCNC, indicates that DCNC acts as an excellent substrate the nucleation, growth, and stabilization of AgNPs [48]. The presence of a polycrystalline circular ring in the electron diffraction (SAED) pattern indicates the crystalline nature of DCNC-AgNPs (Fig. 2D).

During the reduction of Ag^+^, the aldehyde groups of DCNC undergo oxidation at alkaline pH (~ pH 11) after the addition of NaOH, forming a pair of carboxylate (–COO^−^) groups which electrostatically stabilize the developing AgNPs seeds on DCNC substrate [49]. It has been well-known that the aldehydes have a proton attached to the carbonyl carbon which can be abstracted, allowing them to be easily oxidized to form carboxylic acids. According to the literature and as reported by others, NaOH plays a crucial role in initiating the metal nanoparticle growth phase on dialdehyde cellulose by generating alkaline pH which is suitable for the dialdehyde polysaccharide-mediated synthesis of metal nanoparticles due to the deprotonation and the release of sufficient aldehyde groups to reduce metal ions into metal nanoparticles [44, 49]. As observed here, the rate of DCNC-AgNPs formation is very fast, occurring instantly after NaOH addition, which is highly advantageous compared to synthesis using natural polysaccharides or dicarboxy polysaccharides, which can take several hours, and was in accordance to previous reports [49, 50]. This phenomenon was confirmed by the marked decrease in the characteristic carbonyl peak of aldehyde at 1736 cm^− 1^ in the spectrum of DCNC-AgNPs, in addition to the appearance of new peak for C = O groups of carboxylate at around 1598 cm^− 1^ in the FT-IR spectra of AgNPs-DCNC (Fig. 2E). Moreover, the broad absorption band of -OH at 3220 cm^− 1^ in DCNC was shifted to 3281 cm^− 1^ in the FT-IR spectrums of AgNPs-DCNC, possibly due to the interactions of C–OH groups with AgNPs [44] (Fig. 2E).

The X-ray diffraction pattern confirms the presence of characteristic Ag peaks in the biosynthesized AgNPs produced by the DCNC (Fig. 2F). The XRD peaks at 2*θ* of 22.7° corresponds to DCNC, while 2*θ* of 38.48°, 44.38°, and 64.94° are characteristic of (111), (200), and (220) FCC planes of Ag in DCNC-AgNPs (JCPDS File No. 01-1174). According to Scherrer’s equation, the average size of the nanoparticles is 6.97 ± 2.34 nm.

The XPS survey spectrum of DCNC-AgNPs (Fig. 2G) revealed the presence of a C1s peak at 285.38 Ev and an O 1s peak at 532.58 eV. The high-resolution scan of Ag 3d (Fig. 2H) has shown two peaks at 366.6 eV and 372.6 eV, which corresponds to the Ag (0) 3d_5/2_ and 3d_3/2_, respectively, suggesting the formation of metallic Ag atoms. Mostly, the metallic Ag is exposed at 368.1 eV (Ag 3d_5/2_) and 374.1 eV (Ag 3d_3/2_), with a 6.0 eV slitting of the Ag 3d doublet [51]. However, compared to metallic Ag, the peaks in the AgNPs-DCNC are relocated at 1.5 eV to the positive side due to the binding of DCNC, while a splitting of 6.0 eV was retained. Thus, indicating successful DNCN-mediated in situ synthesis of AgNPs. The schematic of the DCNC-AgNPs synthesis reaction is shown in Fig. 2I.

#### Fabrication and characterization of pickering emulsions

**Fig. 3:**
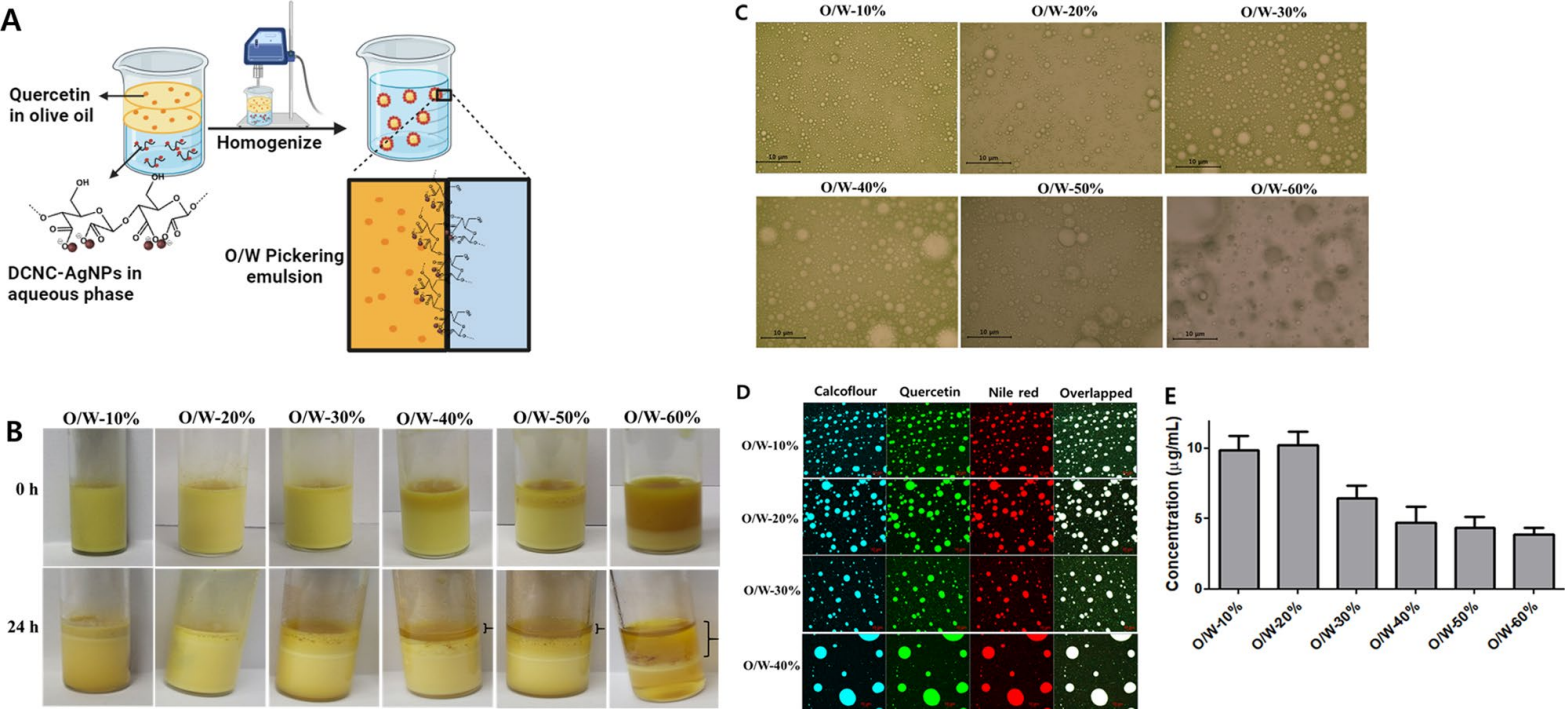
Preparation and characterization of Pickering emulsion at varying O/W ratio. (**A**) Schematic of Pickering emulsion fabrication (Created with BioRender.com (**B**) Representative images where bracketed arrow indicates phase separation (**C**) Optical microscopic images (scale bar 10 µM) (**D**) Confocal images (scale bar 10 µM). (**E**) Quercetin concentration in the emulsion phase

The O/W ratio and concentration of nanoparticles are important parameters for the preparation and stabilization of the PEs. Thus, here, the PE formation was optimized by varying the O/W ratio (Table 1), while keeping the concentration of DCNC-AgNPs constant. Figure 3A shows the schematic description of preparing PEs, and Fig. 3B shows the photographic representations illustrating the prepared Qu-loaded PEs that have been stabilized by DCNC-AgNPs, with consideration to the different O/W ratios. Furthermore, it was observed that a distinct bilayer formed within the glass vial when the oil phase concentration ranged from 40 to 60%. Specifically, when the % of oil is increased, the emulsion layer at the bottom diminishes while the oily layer at the top becomes more prominent.

**Table 1:** Observation of PEs prepared by varying O/W ratios

Sample name	O/W %	Observations
O/W-10%	10	O/W PE was formed without phase separation after 24 h
O/W-20%	20	O/W PE was formed without phase separation after 24 h
O/W-30%	30	O/W PE was formed with little phase separation after 24 h
O/W-40%	40	O/W PE was formed with significant phase separation after 24 h
O/W-50%	50	O/W PE was formed with significant phase separation after 24 h
O/W-60%	60	No formation of O/W PE

The phase separation of the emulsions, shown in Fig. 3B, is a measure of PE stability. The ratios of the creaming phase divided by the whole sample volume were recorded [52]. Considering the lower density of the oil phase, the oil droplets tend to rise to the top of the sample. Oppositely, the aqueous phase tends to accumulate at the bottom when instability occurs [53]. Following 24 h of preparation, the obtained results evidenced the stability of the two PEs, i.e. 10% and 20% O/W, where no phase separation was observed. Notable phase separation were observed from 30 to 50%, with no emulsion layer, but a settled aqueous layer at the bottom, in 60% O/W PE. In addition, clear instances of oil accumulation at the top of the PEs prepared by 40–60% O/W ratio, an observation related to its lower stability. The 20–50% O/W PEs were found to be stable even after 30 days of preparation (Supplementary Fig. S2).

The optical microscopic pictures (CX31, OLYMPUS, Japan), captured immediately after preparation, demonstrated the effective ability of DCNC-AgNPs to create uniform oil-in-water PEs within the oil phase range of 10–50% (O/W ratios), as depicted in Fig. 3C. In contrast, no instances of O/W PEs were discovered when the O/W ratio of 60%. Furthermore, it was noticed that there was an increase in the coalescence of PEs in optical microscopic images as the O/W ratio was increased to 40% and 50%. Thus, PEs produced with 10%, 20%, and 30% O/W ratios had smaller droplet sizes, which remained stable throughout the analyzed period, thus indicating a high resistance to physical instabilities, such as coalescence and Ostwald ripening [54]. As the volume proportion of oil increases, there is a corresponding increase in the area requiring stabilization, resulting in the formation of larger droplets and less stable emulsions. Similarly, a low concentration of particles may be insufficient to adequately cover the entirety of the interfacial area. This phenomenon is often mitigated by the concurrent increase in droplet size and decrease in emulsion stability [54].

Confocal images of the PE, co-loaded with Qu and Nile red, showed the successful encapsulation of both Qu and Nile red in the PE at O/W ratios from 10 to 50% (Fig. 3D). However, the size of PEs increased to a greater extent at O/W-50%, possibly due to coalescence of multiple emulsion droplets into a single event [55], as observed in optical microscopic images. Thus, indicating the instability of PEs. Furthermore, due to the capability of calcofluor white being a fluorophore that binds to carbohydrate residues, it was employed to stain DCNCs-AgNPs. The interpenetration of DCNC-AgNPs in the oil phase was observed through the presence of calcofluor white-stained DCNC-AgNPs in both the oil and water phases of the PEs. This phenomenon is potentially feasible as it has been documented in earlier studies that both CNCs and modified CNCs possess amphiphilic characteristics, which can be attributed to the existence of a hydrophobic edge plane inside the CNC structure [56].

Additionally, it was observed that the highest concentration of Qu was detected in the emulsion phase of O/W-10% and O/W-20% after 24 h of PE development, as depicted in Fig. 3E. These findings suggest that the optimum O/W ratios to produce stable PEs are 10% and 20%.

###  Fabrication and characterization of pickering emulsion-reinforced chitosan films (CS-PE) preparation

**Fig. 4:**
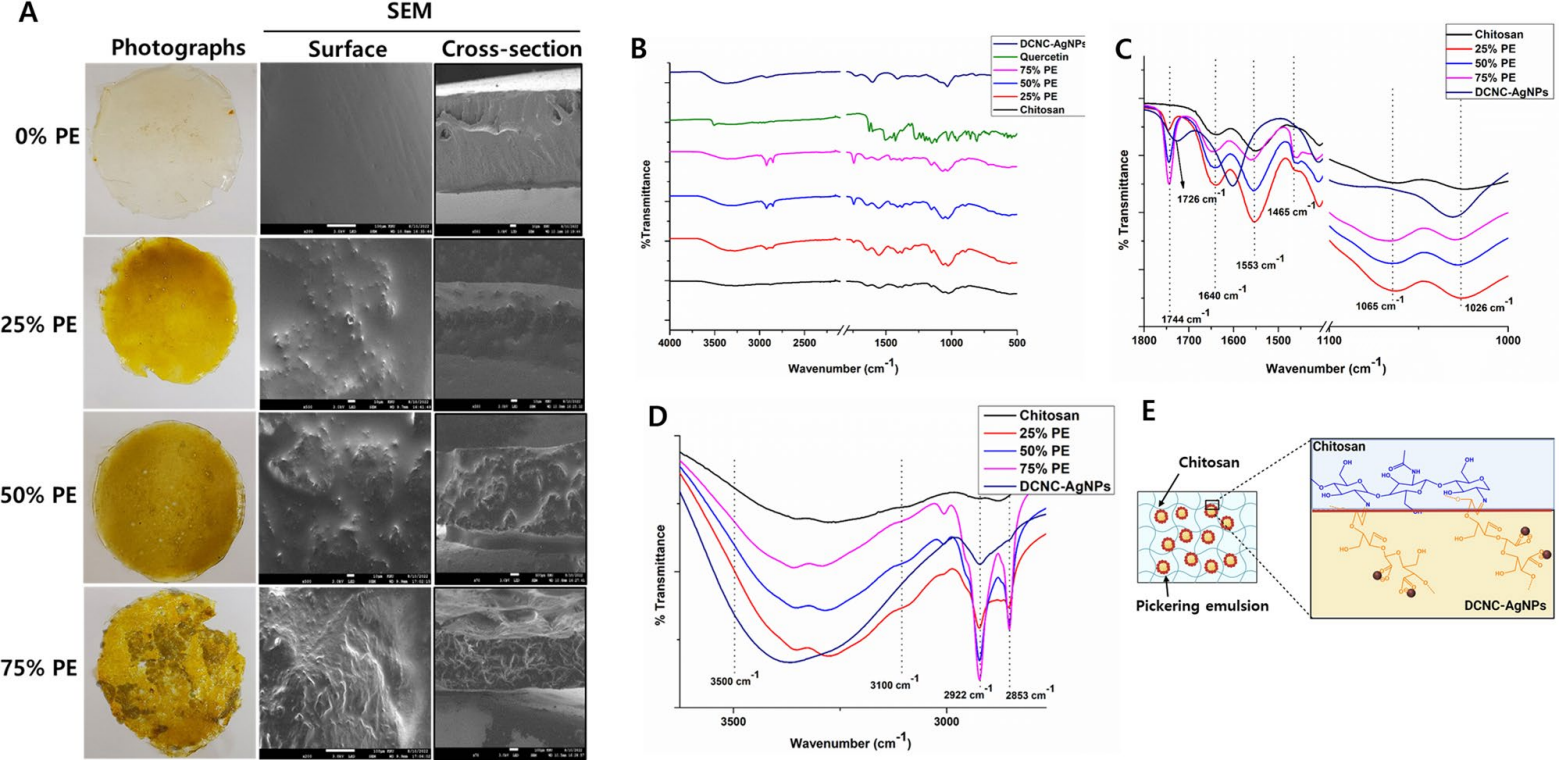
Images and FT-IR spectrum of CS films infused with varying ratios of Pickering emulsion (v/v) (**A**) Representative photographs and SEM images (surface and cross-section) (3 kV, x500, and scale bar 10 µM). (**B**) FT-IR full spectrum (from 4000 to 500 cm^− 1^). (**C**) FT-IR spectrum (from 1800 to 1000 cm^− 1^). (**D**) FT-IR spectrum (from 3700 to 2500 cm^− 1^). (**E**) Schematic of reaction between DCNC-AgNPs stabilized Pickering emulsion and CS films (Created with BioRender.com)

The relationship between the microstructure of films and their physical properties is widely recognized. Figure 4A displays the photographic representation, as well as the surface and cross-sectional scanning electron microscopy (SEM) images of the CS-PE films. The film composed of pure CS exhibited a uniformly smooth surface and a dense and homogenous cross-sectional structure, which can be attributed to the entangled arrangement of CS molecules, consistent with previous findings [56]. However, when the PEs were added to the CS films, the surface gradually became rough with a uniform appearance of granulated structures on the surface of the films. The roughness of the surface increased with increasing PE ratios from 25 to 75%. Similarly, in cross-section SEM images, the inter-penetrated network of the CS-PE films increased with increasing PE ratios due to self-crosslinking between the dialdehyde group in DCNC-AgNPs and the amino group of CS. In addition, at 75% PE, many evenly distributed loose fibrous structures were observed in CS-PE films, possibly due to the more crosslinking between DCNC-AgNPs and CS.

The crosslinking between DCNC-AgNPs and CS was confirmed by FT-IR analysis. Figure 4B-D shows the FT-IR spectra of the CS-PE films with different PE ratios. The C-O stretching vibration of the glycosidic linkages within the CS molecule was observed at 1065 cm^− 1^ and 1026 cm^− 1^ [57](Fig. 4C). The disappearance of aldehyde carbonyl stretching peak at 1726 cm^− 1^ (characteristically assigned to the DCNC), after the addition of PEs to the CS is possibly due to the involvement of aldehyde groups in DCNC in the imine bond formation with the amine groups of CS [28]. In addition, the peaks at 1640 cm^− 1^ (-CO stretching in the amide I) and 1553 cm^− 1^ (N–H bending of the primary amine) were observed in CS spectrum [28, 58] (Fig. 4C). These Peaks showed slight shifting in the CS-PE films along with concurrent decrease in the peak intensity with increasing PE content, possibly because a Schiff base bond was formed between the reactive aldehyde groups of DCNC-AgNPs and amino groups of CS which leads to the change in the vibrational frequency due to the change in their bond length [28]. According to the literature, the vibration of the imine bond itself is relatively weak and should be found around 1630–1640 cm^− 1^ [59]. Thus, the imine formation between DCNC-AgNPs and CS is likely to be overlapped with existing bands of CS at 1640 cm^− 1^. In addition, a new peak at 1465 cm^− 1^ appeared which was found not visible in either of the CS and DCNC-AgNPs spectrums, indicating the possibility of the amide II bond (C-N-C = O vibrational stretching) formation between the DCNC-AgNPs and CS. With the increasing content of PEs, the peaks at 1744 cm^− 1^, 2922 cm^− 1^, and 2853 cm^− 1^ were increased which can be attributed to the C = O stretching, aliphatic C-H stretching frequency from CH_2_ and CH_3_, respectively, from olive oil present in the PEs [60]. The broad spectrum from 3100 to 3500 cm^− 1^ was from the OH group which was overlapped with NH_2_ stretching vibration in CS which also showed shifting and decrease with increasing PE content (Fig. 4D). Figure 4E shows the possible schematic of the reaction between DCNC-AgNPs-stabilized PE and CS.

**Fig. 5:**
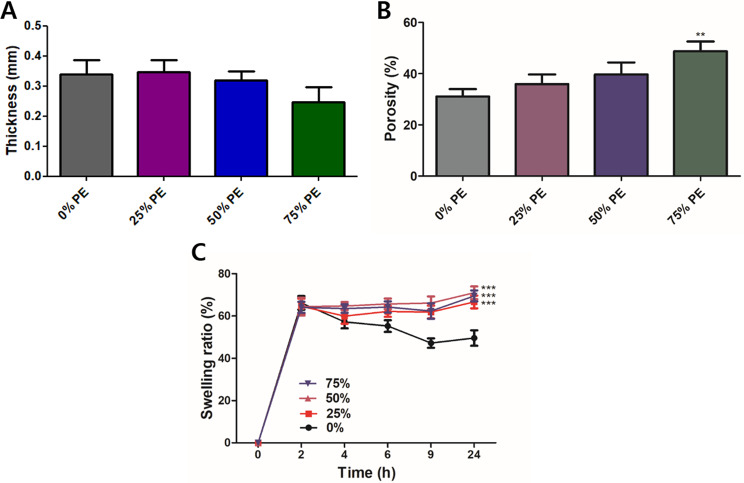
Physical characterization of CS films reinforced with varying ratios of PE (v/v) (**A**) Thickness. (**B**) Porosity. (**C**) Swelling ratio. Values were expressed as mean ± SD, **P < 0.01, ***P < 0.001 vs. 0% PE

Further, non-significant differences in the thickness of the pure CS film, 25% PE-loaded CS films, and 50% PE-loaded CS films were observed (Fig. 5A). However, the thickness of the films appeared to decrease, though non-significant, when the PE content was increased to 75%, possibly due to the interference caused by PE in the formation of CS films by interfering in the alignment and bonding of CS molecules. This result is also in corroboration with SEM images where a uniformly distributed loose fibrous network with plenty of voids was observed in 75% PE-loaded CS film. In addition, an increase in porosity was also observed with an increase in PE content with maximum porosity in 75% PE-loaded CS film (Fig. 5B), which also confirms the SEM results. The porosity of the biopolymer matrix plays a crucial role in the healing process due to its capacity to facilitate cell filtration, promote high permeability, and enable the flow of oxygen and nutrients [61]. Although 75% PE-loaded CS films showed maximum porosity, the photographs showed that 75% PE-loaded CS films were non-homogenous, which is not desirable (Fig. 4A). In addition, it was observed that swelling ratio was also increased when PEs were added to the CS film (Fig. 5C). In general, swelling ratio increases with increase in porosity [62]. However, although the porosity increased with increasing PE content, the swelling ratios were almost similar in 25%, 50%, and 75% PE ratios. This could be possible due to the inter-penetrated network within the matrix between DCNC and CS or partial crosslinking between the AgNPs and the hydroxyl and amine groups in the CS-DCNC chains [63]. Based on these results it can be suggested that 50% PE-loaded CS films might be suitable as wound dressing material, as it can load maximum PE to the films without affecting its integrity.

### Effect of glycerol concentration on the mechanical properties of CS-PE films

Glycerol is often added to pharmaceutical films as a plasticizer to improve their flexibility and mechanical properties. Glycerol plays a major role in the structural arrangement of CS films that influences the molecular mobility, mechanical, barrier, and structural properties of the film [64]. As shown in Table 2, the thickness of the CS-PE films is directly proportional to the glycerol content. Further, the CS-PE films without any glycerol have the highest modulus (i.e., 288.69 ± 89.07 Pa), which decreases concomitantly with increasing glycerol %. The Young’s modulus was lowest and became almost constant in CS-PE-25% Gly and CS-PE-35% Gly films. This drastic drop in the modulus of the CS-PE films, containing even 2.5% glycerol, is attributed to the formation of hydrogen bonds between CS molecules and glycerol, resulting in weak intramolecular interaction between the CS chains, lowering the modulus. Similar findings were also reported by Kusmono et al., who found that 30% glycerol decreased the modulus of CS/CNC biocomposite films [65]. Wound healing films refer to materials that have a high stiffness or resistance to deformation and thus are less prone to deformation or stretching under the forces encountered during the healing process. Therefore, films with low modulus indicate a decrease in the stiffness of the films which could be beneficial in wound healing applications as they provide higher confortability and higher strength of adhesion [66].

**Table 2:** Mechanical properties of CS-PE films prepared ar varying glycerol content

Sample Name	Thickness (mm)	Young’s Modulus (MPa)	Elongation at break (%)
CS-PE-0% Gly	0.75 ± 0.15	287.97 ± 89.07	2.38 ± 0.35
CS-PE-2.5% Gly	0.62 ± 0.08	0.77 ± 0.24	28.625 ± 7.72
CS-PE-10% Gly	0.92 ± 0.08	0.15 ± 0.03	32.67 ± 10.17
CS-PE-25% Gly	1.82 ± 0.18	0.04 ± 0.02	44.5 ± 13.78
CS-PE-35% Gly	2.6 ± 0.1	0.03 0.01	73.45 ± 10.77

**Fig. 6:**
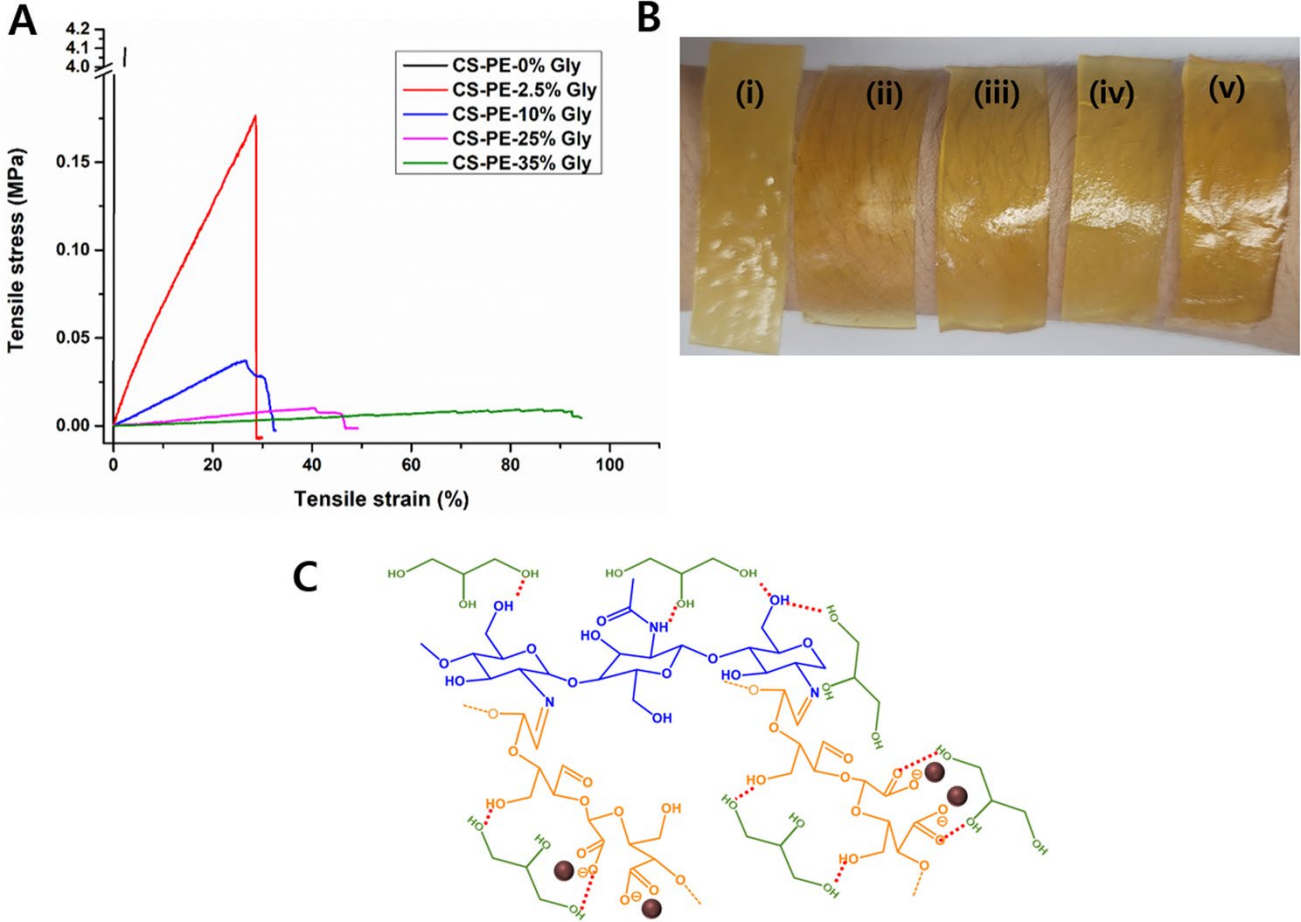
(**A**) Stress and strain curve (**B**) Representative photographs of (i) CS-PE-0% Gly, (i) CS-PE-2.5% Gly, (iii) CS-PE-10% Gly, (iv) CS-PE-25% Gly, and (v) CS-PE-35% Gly films. (**C**) Molecular interaction of glycerol with CS-PE films to impart plasticizing effect

In contrast to modulus, the elongation at break drastically increased in the order of 2.5% < 10% < 25% < 35% glycerol content (Table 2). The elongation at break quantifies the capacity of a material subjected to tensile forces to endure stretching or deformation before failing. In the context of wound healing films, moderate to high elongation at the break would confer a significant advantage by allowing the film to effectively conform to the wound bed’s irregularities and accommodate the skin’s elongation during bodily movements and other physical exertions. The use of a wound healing film with excessive stiffness or rigidity may cause discomfort during wear and may restrict movement or physical activity, thereby potentially slowing the rate of healing [67]. Thus, for wound healing films, a moderate to high elongation at break is generally desirable to ensure that the films display desirable conformability and flexibility. Here, it was observed that the elongation at break of CS-PE-25% Gly and CS-PE-35% Gly were in accordance with some of the commercially available wound dressing films making them suitable wound dressing material (Table 2) [68]. Minsart et al. [68], determined mechanical parameters for some of the frequently used commercial wound dressings and found that Young’s modulus, total elongation, and ultimate stress may largely vary between these commercially available wound dressings based on their fabrication methods. However, they showed that the dressing with the lowest Young’s moduli but the highest total elongation signifies that they are very flexible. In contrast, the highest Young’s modulus and the lowest total elongation render stiffness to the dressings. Since we found that our CS-PE hydrogel films have low Young’s moduli and high total elongation, we could suggest the flexibility of the CS-PE hydrogel films. The stress and strain curve and representative photographs of CS-PE films with varying Gly content are shown in Fig. 6A & B, respectively. The increases in the flexibility and thickness of CS-PE films with increasing glycerol content, which is consistence with previous literature [69]. Since CS-PE films with 0% glycerol were too stiff to be used for wound healing applications, CS-PE 0% glycerol films were removed from further studies. The molecular interaction between glycerol and CS-PE films is shown in Fig. 6C.

### Effect of glycerol concentration on the physical properties of CS-PE films

**Fig. 7:**
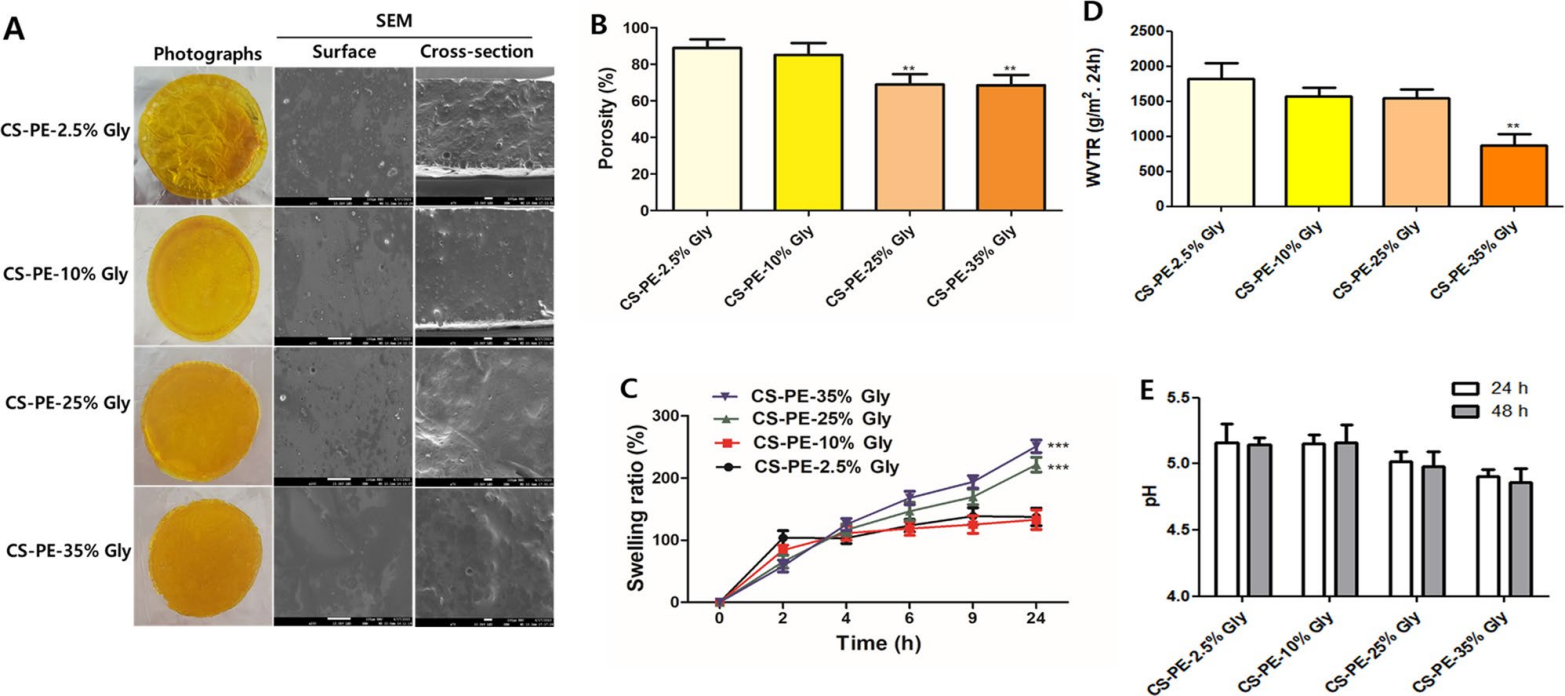
Characterization of CS-PE films with varying glycerol content (**A**) Representative photographs and SEM images (surface and cross-section) (3 kV, x500, and scale bar 10 µM) (**B**) Porosity (**C**) Swelling ratio (**D**) Water vapour transmission rate and (**E**) Dressing pH. Values were expressed as mean ± SD, **P < 0.01, ***P < 0.001 vs. CS-PE-2.5% Gly

Figure 7A shows the photographs and SEM images (surface and cross-sectional) of CS films prepared by adding 50% PE and varying amounts of glycerol from 2.5 to 35%. The photographs of CS-PE at all concentrations of glycerol show a smooth and uniform surface without holes, cracks, and insoluble particles, suggesting the good miscibility and compatibility of glycerol with PG. However, the microstructure of the CS-PE films, as seen in the cross-sectional SEM images, shows that the roughness of the films due to the incorporation of the PE was decreased and the smoothness increased in the CS-PE films with increasing glycerol concentration (Fig. 7A). Thus, revealing more compactness and uniform microstructure at higher glycerol concentration [70]. Further, higher concentrations of glycerol tend to reduce the film porosity (Fig. 7B), possibly by increasing the film’s density and reducing void spaces [70].

Moreover, non-significant changes in the swelling ratios of CS-PE films with 2.5% and 10% glycerol content were observed. However, the swelling ratio significantly increased with increasing glycerol content at 25% and 35% glycerol content (Fig. 7C). These results are consistent with the previous studies [71]. The swelling ratio of films is often associated with the polymer’s ability to absorb and retain water. Although the films with strong network structures between polymer chains result in resistance to the rapid penetration of water, the presence of glycerol can reduce the intermolecular forces between polymer chains, thus increasing the swelling capacity of the films. Therefore, despite of strong network in the CS-PE films fabricated in this study, high glycerol addition possibly increased its swelling ratio, suggesting that the as-prepared biocomposite film can create a moist environment surrounding the wound and effectively absorb fluids from the wound.

A controlled suitable moist environment is essential for effective wound dressings to prevent excessive wound dehydration and accumulation of exudate, facilitate wound re-epithelialization, and prevent scar formation [72]. Since WVTR provides information about the moisture management properties of the dressing materials, it is an important indicator for wound-dressing evaluation. Glycerol content also had a significant impact on the WVTR of CS-PE films. The WVTR decreased with increasing glycerol content, possibly due to the increase in flexibility and thickness which can improve barrier properties against moisture (Fig. 7D). The water evaporation rate for normal skin is 204 g/m^2^ per day [73]. If the WVTR of a material is lower than that of normal skin, tissue becomes dried out, while exudate between the wound and the covering results in an infection. Therefore, materials for wound dressing should have a higher WVTR value than normal skin [74]. Since the WVTR of all the films was above the water evaporation rate for normal skin, all the films were suitable as wound dressing material.

The measurement of pH in wound dressings is a crucial factor as it serves the purpose of not only regulating infection at the wound site but also expediting the growth of fibroblasts. Ideally, it is desirable for a wound dressing to maintain a slightly acidic environment on the surface of the wound. This is because The pH of normal healing wounds is in the range of 5.5–6.5 during the healing phase. However, in non-healing infected wounds, the pH will be above 6.5. By doing so, the wound healing process can be expedited when compared to a neutral or alkaline environment [33]. The dressing pH values of CS-PE films are depicted in Fig. 7E, and they fall within the range of 4.9 to 5.16, which is in agreement with previous literature [33]. Based on the findings, it can be inferred that the CS-PE film possesses the capability to create an acidic environment on the surface of wounds, hence promoting cell proliferation and fibroblast development.

### Release of Qu and silver ions from CS-PE films and skin permeation study

**Fig. 8:**
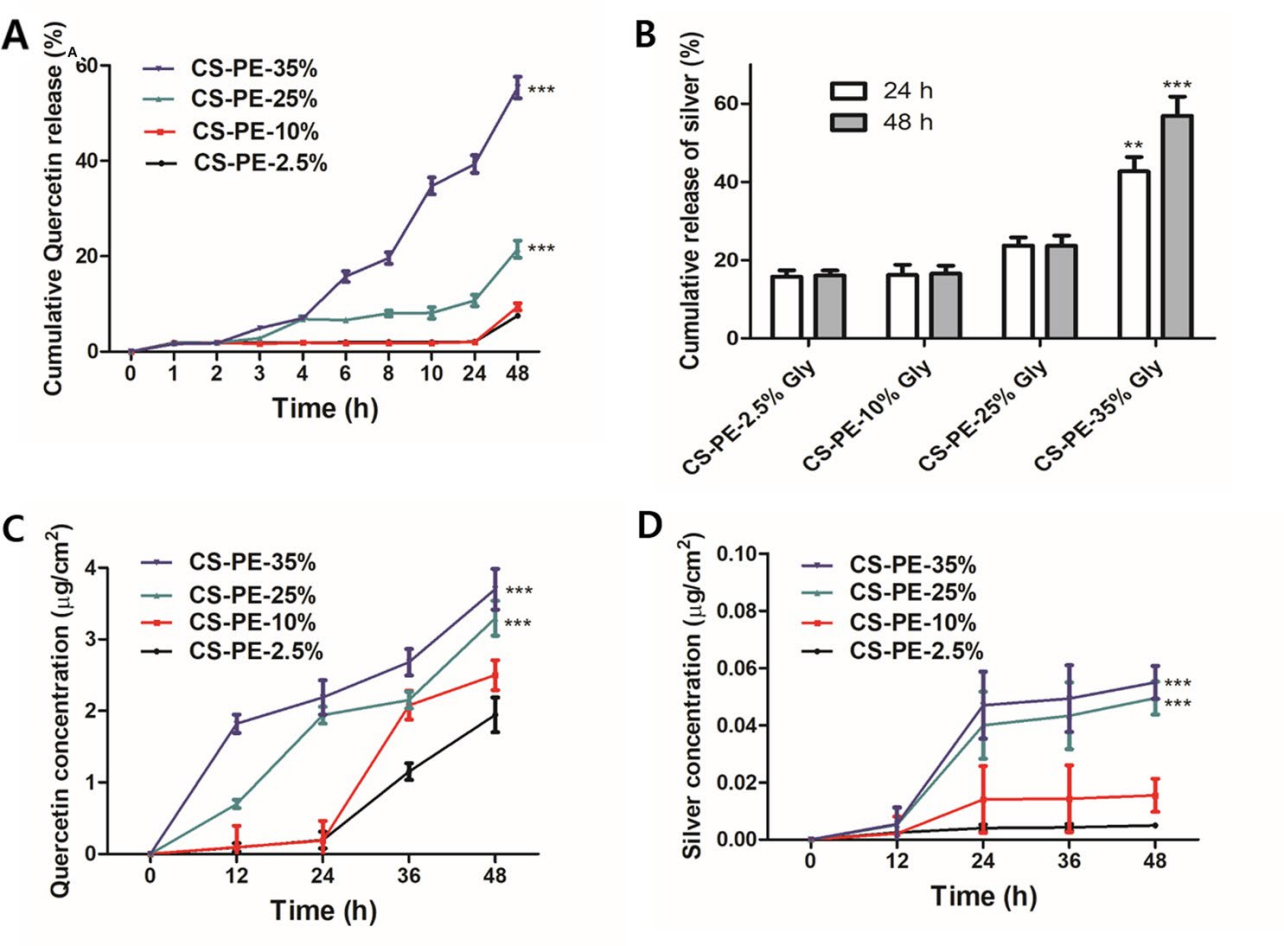
Time-dependent drug release from CS-PE films with varying glycerol content (**A**) Release of quercetin. (**B**) Release of silver ions. (**C**) Skin permeation of Quercetin. (**D**) Skin permeation of silver. Values were expressed as mean ± SD, **P < 0.01, ***P < 0.001 vs. CS-PE-2.5% Gly at respective times

Hydrogels could control the release of Ag^+^ from AgNPs and loaded drugs in a gradient manner owing to their physico-chemical properties, such as absorption, swelling, and degradation [75, 76]. Further, the plasticizing effect of glycerol allows for tighter packing of hydrogel film-forming materials, resulting in a more compact film structure with reduced pore volume. This decreased porosity can restrict drug diffusion, leading to slower drug release rates. Since the wound dressings are recommended to be changed within 12 to 48 h [77], we observed the release profile of Ag^+^ and Qu from CS-PE hydrogel films with varying glycerol contents for 48 h only. Interestingly, in the current study, it was observed that while increased glycerol content reduces film porosity (Fig. 7B), it increases Qu and Ag^+^ release rates (Fig. 8A&B). This could be possible because the presence of glycerol might enhance the mobility of Qu and Ag^+^ within the film matrix [78], allowing them to diffuse more freely. This increased mobility can overcome the restriction imposed by reduced porosity, resulting in the accelerated release of Qu and Ag^+^. Additionally, glycerol can increase the solubility of Qu and Ag^+^ in the film matrix [79], enhancing release kinetics. Moreover, it is also possible that due to the increase in swelling ratio with an increase in glycerol content, as shown in Fig. 7C, the diffusion of the drugs to the outside solution was increased. Thus, it can be concluded that the glycerol content has a significant impact on drug release which can be exploited to optimize CS film formulations for wound healing applications. Here, the result shows that higher glycerol concentrations can increase drug release rates. Thus CS-PE-35% Gly was selected for further experiments.

The In vitro permeation of Qu and silver was studied using artificial skin, Strat-M ® membrane, as they are designed to closely mimic the properties of human skin and are often used as an in vitro model for transdermal drug delivery and permeation studies [80]. It was observed that cumulative Qu concentration in the receiving chamber was steadily increased with glycerol content (Fig. 8C), indicating that the presence of glycerol in the films might assist in the permeation of Qu [81]. Moreover, higher release of Qu in the presence of glycerol might also be the reason for higher skin permeation. Further, Ag^+^ was also observed in the receiving chamber which also significantly increased with increasing glycerol percentage (Fig. 8D). Previous in vivo and ex vivo studies also showed the penetration ability of silver (as Ag^0^ or Ag^+^) through the skin [82, 83]. Furthermore, Nešporová et al. also observed that the skin permeation of silver-containing creams is increased with permeation enhancers, such as glycerol [84].

### In vitro cytocompatibility of CS-PE films

The preceding characterizations of CS-PE films implied that the CS-PE-35% Gly films had the most suitable physical and mechanical characteristics to be used as a wound dressing material. Thus, all the CS films for wound healing assessment were prepared with 35% Glycerol, i.e. blank CS, CS-Qu, CS-blank PE, and CS-PE-Qu films were used as representatives to assess the wound healing potential. In HaCat cells, the cell viability was significantly less in CS extract 1 and significantly higher in extract 3 of CS-blank PE and CS-PE-Qu. Moreover, the growth of HDFa cells was significantly reduced when treated with extract 1 of CS film while no significant changes in cell viability of HDFa cells were observed in all the tested extracts of CS-PE-Qu films, compared to the control (Supplementary Fig. S3). The observed paradoxical outcome can be attributed, at least in part, to the variations in chemical compositions and physical properties of the CS samples under investigation [85–88]. Key attributes of CS encompass its molecular weight, degree of deacetylation, crystallinity index, monomeric unit count, and composition which can affect the viscosity, water retention capacity, and charge density of the CS [89]. In agreement with our result, Dara et al. also showed that CS-AgNPs composites were non-toxic to L929 fibroblast cells [90]. Levi-Polyachenko et al. showed that the cytotoxicity of CS-AgNPs films on keratinocytes and fibroblasts depends on the size and amount of AgNPs in the CS-AgNPs composite [91]. In Our investigation, the AgNPs alone or in combination with Qu-containing PEs reduce the cytotoxic effect of CS films on the HaCat and HDFa cells.

### Effect of CS-PE films on the cell migration potential and collagen secretion

**Fig. 9:**
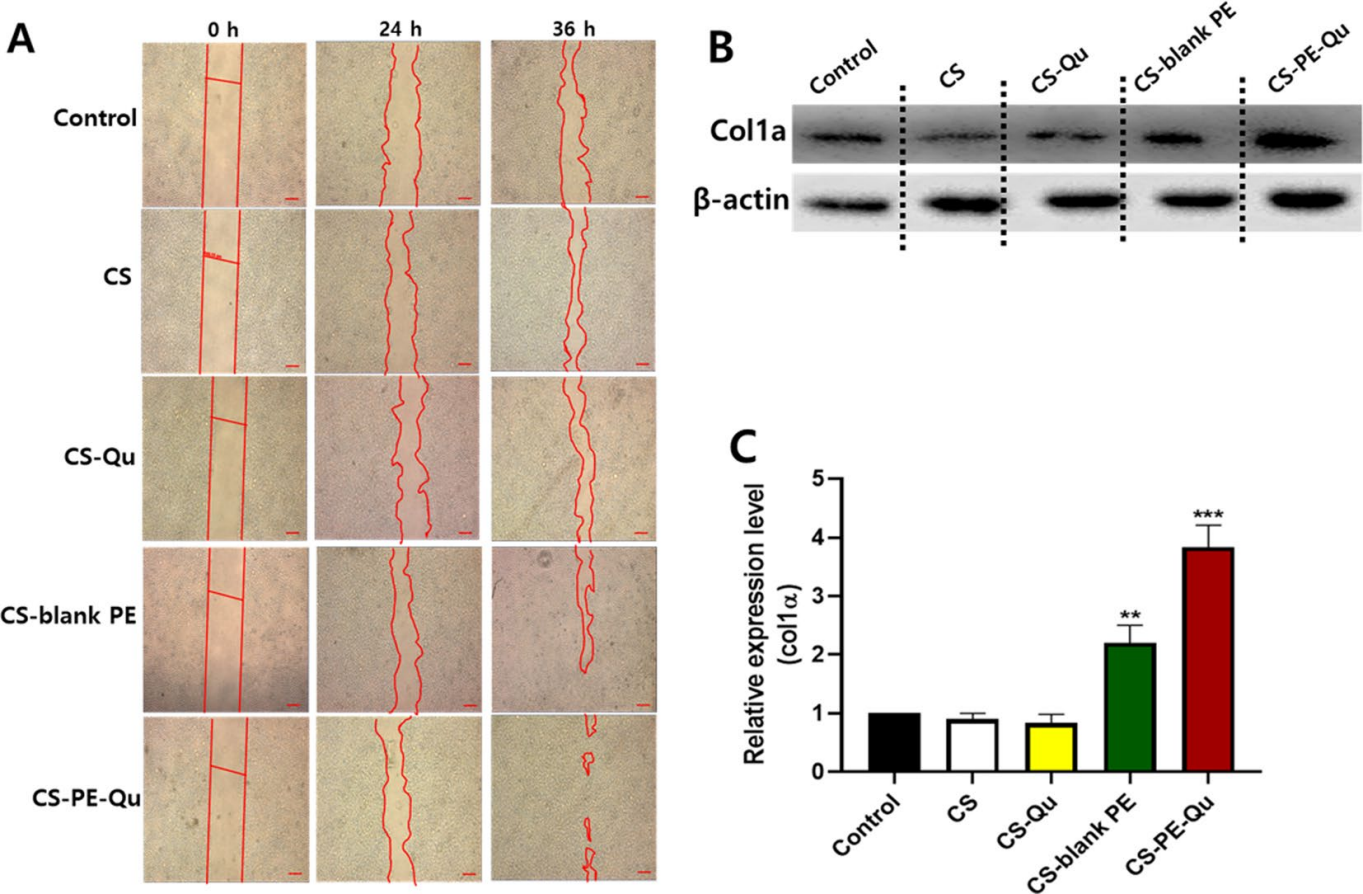
In vitro assays (**A**) Cell scratch assay showing pattern of cell migrations in the scratch area after treatment with CS, CS-Qu, CS-blank-PE, and CS-PE-Qu films on HaCat cells (scale bar 200 µM). The scratched area is marked by red lines (**B**) Representative western blot and (**C**) Densitometry analysis with respect to *β*-actin of collagen production by HDFa cells after treatment with CS, CS-Qu, CS-blank-PE, and CS-PE-Qu films. Quantitative densitometric analysis of protein was performed by using Fusion FX software. The results were normalized with *β*-actin expression. Data are shown as the mean ± SD of three independent experiments. *P ≤ 0.05, **P ≤ 0.01, ***P ≤ 0.001 vs. control

Enhancement of epidermal migration is among the crucial criteria of an ideal wound dressing material [92]. To evaluate the time-dependent in vitro migration of cells, scratch assays are commonly used in response to various stimuli [93]. In this study, the migration of human HaCaT keratinocytes and HDFa cells was investigated after treatment with extracts of CS, CS-Qu, CS-blank PE, and CS-PE-Qu films. It was noted that post-24 h treatment, the cells treated with CS and CS-Qu extracts did not show significant differences in migration, compared to the control group. While, at the same time point, the cellular migration increased in cells treated with CS-blank PE and CS-PE-Qu extracts, compared to control cells (Fig. 9A). Further, after 36 h of treatment, the cellular migration was maximum in CS-PE-Qu extract treated cells, suggesting significantly faster wound healing abilities of CS-PE-Qu films, possibly due to the synergistic effect of Qu and AgNPs.

In contrast, CS-PE-Qu films showed no visible effect on the migration of HDFa cells after 24 h and 36 h of treatment (Supplementary Fig. S4), which could be possible due to the challenges posed by the elongated cellular morphology of the HDFa cells in observing the cellular migration in two-dimensional microscopy. Nevertheless, a significant increase in Collagen 1α (Col1 α) production was found in treated HDFa cells when treated with CS-blank PE and CS-PE-Qu films (Fig. 9B&C). This corresponds with the re-epithelialization phase of wound healing and reflects the function of fibroblasts in mediating the secretion of extracellular matrix to facilitate collagen production [94]. Liu et al. [95]; however, observed that citrate-coated AgNPs with a mean diameter of 10 nm decreased collagen production in mouse fibroblast cells, which also resulted in decreased viability of fibroblast cells. The increased collagen production is possibly due to the use of biocompatible methods of AgNPs synthesis using DCNC, and also due to the synergistic effect of Qu. In agreement with this result, increased collagen production by dermal fibroblast in response to AgNPs and flavonoids has been previously reported by Bhubhanil et al. [96], where they used a toxic chemical-free synthesis method for AgNPs. Thus, based on the cell proliferation, migration, and collagen production assays, it can be concluded that the biocompatible CS-PE-Qu films may aid in the proliferation and remodeling of cells if used as a wound dressing material.

### In vivo wound healing assessment of CS-PE films

**Fig. 10:**
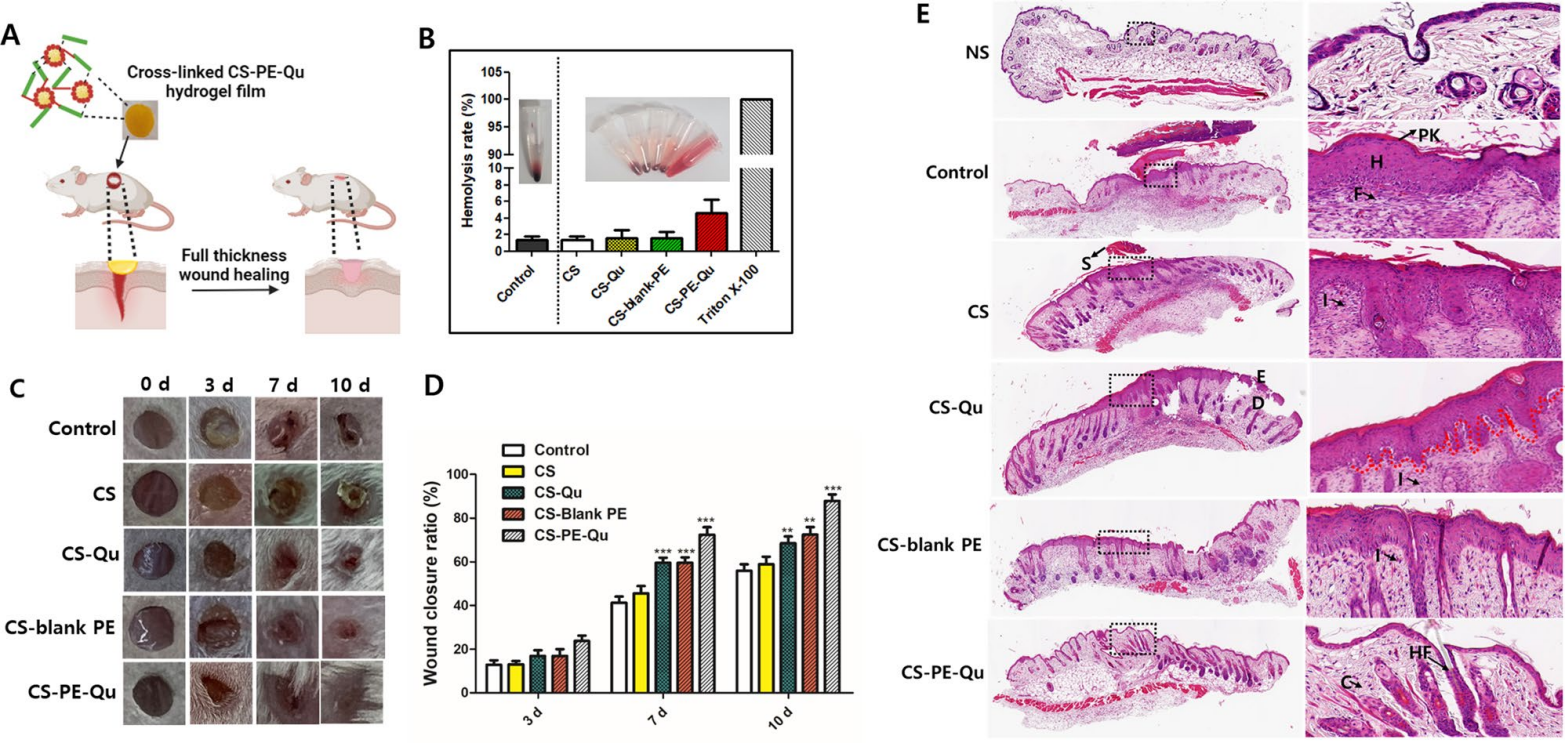
In vivo wound closure assays. (**A**) Schematic diagram of mice model with full-thickness skin wound repaired by CS-PE-Qu hydrogel film (**B**) Hemolysis rate and hemolysis test digital images (inset) of control, CS, CS-Qu, CS-blank PE, CS-PE-Qu films, and positive control (from left to right) (**C**) Representative digital photographs of wounds treated with medical gauze (control group), CS, CS-Qu, CS-blank PE, and CS-PE-Qu films at different time points (**D**) The wound closure ratio at different time points (*n* = 4). (**E**) H&E staining images of the normal skin (NS) and wound tissues treated with CS, CS-Qu, CS-blank PE, and CS-PE-Qu films on day 10. The right panel is enlarged images of dotted black boxes in the left panel. The red dotted lines show re-epithelization, S: scab, E: epidermis, D: dermis, H: hyperplastic, PK: parakeratosis, F: fibroblast, HF: hair follicle, C: collagen, I: inflammatory cells. Values were expressed as mean ± SD, *P < 0.05, **P < 0.01, ***P < 0.001 vs. controls at their respective time point. ^†^*P* < 0.05 and ^††^*P* < 0.01 vs. CS-blank PE at respective time points

The schematic description of mice full thickness wound repair by CS-PE-Qu hydrogel film is shown in Fig. 10A. For an optimal wound dressing, promising hemocompatibility is an essential parameter that should prevent hemolysis [97]. Here, a hemolytic activity assay was performed to analyze the hemocompatibility of the films. As depicted in Fig. 10B, the supernatants obtained from CS, CS-Qu, CS-blank PE, and CS-PE-Qu are almost clear with no visible hemolysis of red cells. In contrast, the positive control (i.e., PBS solution containing 0.1% Triton X-100) exhibited a uniformly vibrant red solution due to the hemolysis of red cells. In addition, the quantitative data revealed that the hemolysis rates of all the tested samples were below the safety threshold of 5% [97], indicating favorable blood compatibility of all CS, CS-Qu, CS-blank PE, and CS-PE-Qu films.

An ideal wound dressing can promote skin regeneration and speed up wound closure, suggesting wound closure rate as a key indicator of wound healing. Figure 10C&D shows the digital photographs and the corresponding wound closure ratios of wounds at 3, 7, and 10 days, respectively. The CS-PE-Qu films group exhibited a better wound healing effect than the control (medical gauze), CS, CS-Qu, and CS-blank PE group. Compared with the control group, the healing ratios of the wounds treated with the CS-blank PE films were significantly higher from the 7th day to the 10th day. Similarly, compared with the control group, the healing ratios of the wounds treated with the CS-PE-Qu films were significantly higher from the 3rd day to the 10th day. The CS-Qu group showed no significant improvement in wound healing compared to the control and CS groups, indicating that free Qu dispersed in the CS films have no wound healing potential, possibly due to its limited delivery to wounds. Further, CS-PE-Qu films showed significant improvement in wound closure compared to CS-blank PE films, suggesting the synergistic effect of Qu in wound healing due to increased bioavailability at the site of the wound. On the 10th day, the CS-PE-Qu film groups almost achieved complete wound healing, though some scars remained. Thus, it was suggested that both the CS-PE-Qu films could improve the wound recovery rate, compared to the other groups.

After 10 days of treatment, histological analysis was conducted to examine the wound healing process in detail. The qualitative grading of the lesions was performed on the following five features: epithelisation, epidermal-dermal attachment, angiogenesis, mononuclear leukocytes, and fibroblast reaction. The outcomes of H&E staining are depicted in Fig. 10E.

Almost complete re-epithelisation was observed in the wound treated with CS-PE-Qu, with very mild hyperplastic surface epidermis compared to normal skin (NS). The epidermis of CS-PE-Qu film-treated mice almost resembled the NS epidermis and was completely attached to the underlying granulated dermis where fibroblasts showed an ordered arrangement with the collagen fibers and prominent vertical perpendicular capillaries. In addition, obvious hair follicles deep into the dermis were also seen [98].

However, although the CS-blank PE-treated wounds also showed re-epithelisation from the edges covering a large proportion of the wound, mild to moderate hyperelasticity with parakeratosis was observed in this group with lack of epidermis-dermis attachments at some places. Also, a slightly disordered arrangement of fibroblast in the dermis was observed. The untreated wound (control) and the wounds treated with CS and CS-Qu showed the least re-epithelisation, with thick epidermal layers, high hyperplastic and parakeratosis appearance at some areas of the wound, suggesting that the wounds entered the stage of tissue regeneration. Similar to the CS-blank PE group, epidermis-dermis detachment was observed in these groups at some places. Even though recruited inflammatory cells were found in the wounds of all groups (control, CS, CS-Qu, CS-blank PE, and CS-PE-Qu films), the density of inflammatory cells in the CS-blank PE and CS-PE-Qu film groups was significantly lower than that of the control and CS film groups.

Thus, indicating that the Qu present in the PE reduced the inflammation and assisted in epidermis regeneration, improving the wound healing rate. This could be possible because the CS-PE-Qu films increased the bioavailability of quercetin, indicating that CS-PE-Qu film is a promising material for wound treatment support.

## Conclusion

In summary, an O/W PE was fabricated using DCNC-AgNPs as a stabilizing nanoparticle which can encapsulate Qu in its oil phase. Further, by reinforcing the quercetin encapsulating and DCNC-AgNPs stabilized O/W PE in the CS films, biocompatible CS-PE-Qu films were fabricated for dual delivery of Ag^+^ and Qu. Glycerol increased the flexibility and swelling rate of CS-PE-Qu films, which promised a comfortable application on the skin and are capable of absorbing large amounts of exudates and providing a moist environment for wound healing. Further, glycerol also increased the release of encapsulated Qu and Ag^+^ from the CS-PE-Qu films. In vitro studies showed cytocompatibility, increased HaCat cell migration, and collagen synthesis from HDFa cells post-treatment with CS-PE-Qu films, compared to control, CS, CS-Qu, and CS-blank PE films, indicating a synergistic effect of Ag^+^ and Qu on wound healing. Further, the CS-PE-Qu films exhibited non-hemolysis and resulted in an enhanced wound-healing process without causing any major skin impairments. Thus, suggesting their candidacy for promising wound dressing material to be used in wound care management in clinics.

### Electronic supplementary material

Below is the link to the electronic supplementary material.

Supplementary Material 1

## Data Availability

No datasets were generated or analysed during the current study.
