# Supplementary material for: Accelerated full-thickness skin wound tissue regeneration by self-crosslinked chitosan hydrogel films reinforced by oxidized CNC-AgNPs stabilized Pickering emulsion for quercetin delivery
Source: J Nanobiotechnology. 2024 Jun 8;22:323. doi: 10.1186/s12951-024-02596-0 (PMC11162036; doi:10.1186/s12951-024-02596-0)
Supplement: Supplementary file 1 — Supplementary Material 1 [file 12951_2024_2596_MOESM1_ESM.docx]

**Supplementary information**

**Accelerated full-thickness skin wound tissue regeneration by self-crosslinked chitosan hydrogel films reinforced by oxidized CNC-AgNPs stabilized pickering emulsion for quercetin delivery**

Garima Sharma^a,*^, Jomon George Joy^a^, Ashish Ranjan Sharma^b^, and Jin-Chul Kim^a,*^

^a^Department of Biomedical Science & Institute of Bioscience and Biotechnology, Kangwon National University, Chuncheon 24341, Republic of Korea

^b^Institute for Skeletal Aging & Orthopedic Surgery, Hallym University-Chuncheon Sacred Heart Hospital, Chuncheon-si, 24252, Gangwon-do, Republic of Korea

^*^Corresponding authors: G.S.; J.-C.K.

Tel.: +82 33 250 6561; Fax.: +82 33 259 5645; E-mail: jinkim@kangwon.ac.kr

**1. Supplementary materials and methods**

**1.1. UV-Vis absorbance**

Preliminary characterization for the formation of the DCNC-AgNPs was carried out using UV–visible spectroscopy after diluting the samples 10 times with Millipore water. The spectra of the AgNPs solution were monitored by a UV-Vis spectrophotometer (Jenway, 7315 UV/Vis spectrophotometer) from 250 to 700 nm. Millipore water was used as a blank to adjust the baseline.

**1.2. Dynamic light scattering (DLS)**

Dynamic light scattering (DLS) using a zeta sizer from Malvern (England) was used to calculate the hydrodynamic particle size of the colloidal dispersions. For each sample, 2 ml of the nanoparticle dispersion was added to a quartz cell before being placed in a zeta sizer. All samples' mean particle sizes were calculated using a scattering angle of 90°. Particle size was measured three times for each sample, and the average was calculated.

**1.3.** [**Transmission electron microscopy**](https://www.sciencedirect.com/topics/materials-science/transmission-electron-microscopy)**(TEM)**

[Transmission electron microscopy](https://www.sciencedirect.com/topics/materials-science/transmission-electron-microscopy) (TEM) was performed with a (JEOL, JEM-2100F, Tokyo, Japan). DCNC-AgNPs suspension was dropped on a Formvar-coated copper grid and dried in open air before TEM observation under 120 kV acceleration voltages. In addition, the nanoparticles were also analyzed for HR-TEM images and [SAED](https://www.sciencedirect.com/topics/biochemistry-genetics-and-molecular-biology/electron-diffraction) pattern analysis.

**1.4. Scanning electron microscopy (SEM)**

The surfaces and broken cross-sections of gold-sputtered films were examined by scanning electron microscopy (SEM) (FE-SEM, JEOL, JSM-7900F, Tokyo, Japan).

**1.5. FT-IR**

Lyophilized CNC, DCNC, and DCNC-AgNPs were put through an FT-IR spectrophotometer (Frontier, PerkinElmer, UK) set to attenuated total reflectance mode to examine the stretching of chemical vibrations.

**1.6. ^1^H NMR**

For ^1^H NMR analysis, the samples were prepared using D_2_O. ^1^H NMR spectroscopy was used to analyze the dialdehyde modification of CNC using an NMR spectrophotometer (JEOL JNM-ECZ400S/L1 400 MHz spectrometer, Japan).

**1.7. XRD**

The crystallinity of the DCNC and lyophilized DCNS-AgNPs was determined by X-ray diffraction (using a Philips X'Pert-MPD diffractometer) following the production of DCNC-AgNPs. In a step-scanning mode with a 2 range of 10-80°, the XRD was employed with Cu-K radiation operating at 40 kV and 40 mA. The crystallinity index of CNC and DCNC was calculated using the Segal equation (Alle et al., 2022):

$$CrI=\frac{I_{002}-I_{am}}{I_{002}} x 100$$

Where, I_002_ represents the intensity of the diffraction peak of the main crystal plane (I_002_, 2θ = 22.5°) and I_am_ represents the intensity of the amorphous halo (I_am_, 2θ = 16.4°), respectively.

Scherrer's equation was also used to calculate the size of the AgNPs (Sharma et al., 2022).

$$D=\frac{0.9\lambda}{\beta Cos\theta}$$

where, D = size of the nanoparticle, *β* = full width at half maximum intensity of the peak in radians, and λ = x-ray wavelength.

**1.8. XPS**

Lyophilized DCNC-AgNPs were analyzed with an XPS (Thermo Fisher Scientific K Alpha+ spectrometer, Waltham, MA, USA) to evaluate their elemental makeup and oxidation state, as detailed in prior work. The Al-Ka x-ray source (1486.6 eV) was used to take the necessary analytical readings. Survey values were derived using the peak energy of C1s (284.8 eV). The spectra were collected using an energy step size of 1.0 eV and a pass energy of 200 eV in a constant analyzer energy mode. The pass energy for narrow scans was 50 eV, while the step size was 0.1 eV.

#### 1.9. Confocal laser scanning microscopy

A laser confocal microscope (Carl Zeiss (LSM880 with Airyscan)) was used to verify the formation of O/W emulsion. The DCNC-AgNPs were stained by Calcofluor white before the synthesis of PEs. The hydrophobic fluorophore Nile Red (100 µL of 0.1 mM Nile Red stock) was added along with Qu in the oil phase before the synthesis of PEs. The distribution of the Nile Red and Qu in the oil phase of the PE and the presence of DCNC-AgNPs on the surface of PEs was then observed in a confocal laser scanning microscope.

**Supplementary Table S1.** The aldehyde content in dialdehyde-CNC

| Time (h) | Aldehyde content (mM/g) |
| --- | --- |
| 2 | 0.76 |
| 4 | 3.1 |
| 6 | 4.9 |
| 8 | 6.5 |
| 10 | 6. |

**
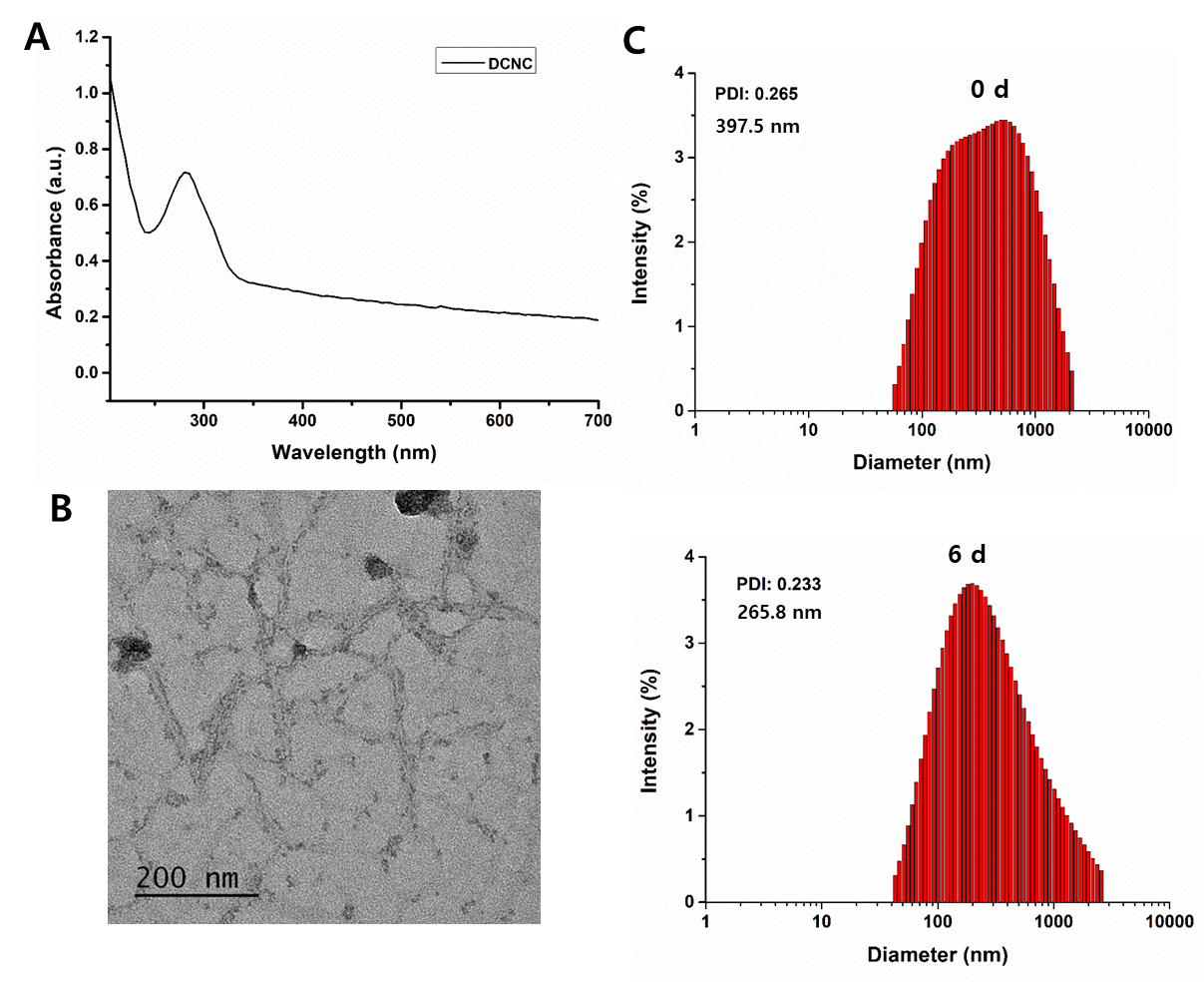
**

**Supplementary Figure S1. (A)** UV-Vis spectra, **(B)** morphology of DCNC, and **(C)** time-dependent hydrodynamic size at day 0 and day 6 indicating the stability of DCNC.

**
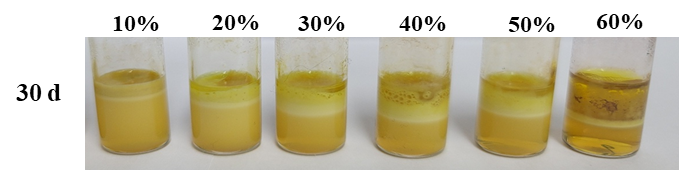
**

**Supplementary Figure S2.** Stability of Pickering Emulsions after 30 days of preparation.

**
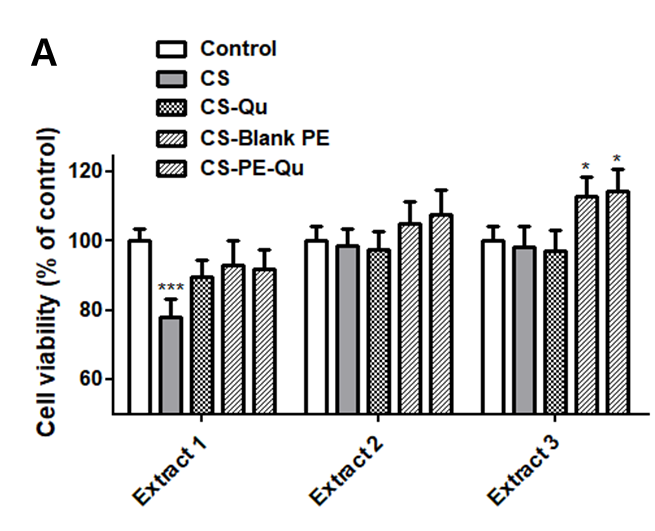
**

**
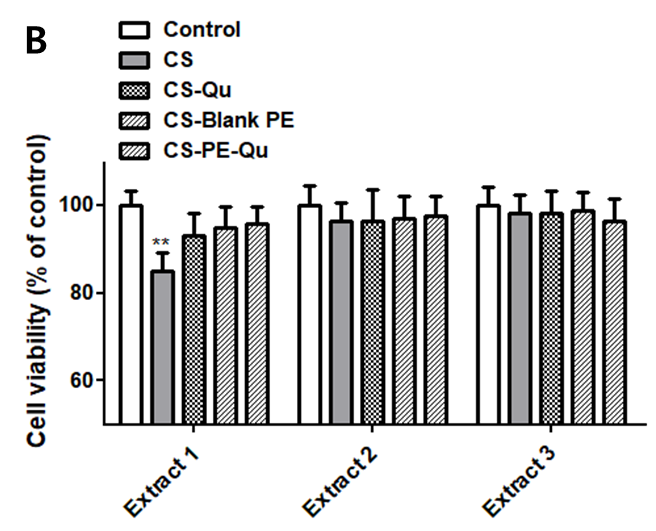
**

**Supplementary Figure S3.** Cell viability assays of CS films on **(A)** HaCat cells **(B)** HDFa cells.


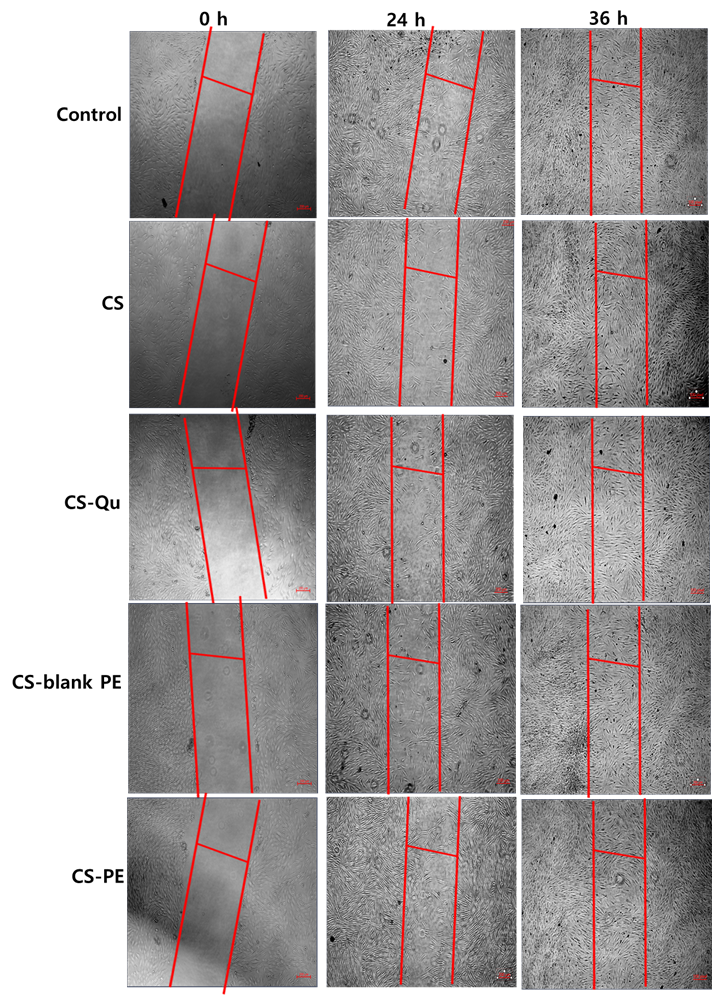


**Supplementary Figure S4.** Cell migration assay after treatment with CS, CS-Qu, CS-blank-PE, and CS-PE-Qu films on HDFa cells.
